# Co‐Creation for Health System Innovation: Developing a Model and an Action Plan for Future Primary Care in Switzerland

**DOI:** 10.1111/hex.70927

**Published:** 2026-10-22

**Authors:** Anna Romanova, Andreas Balthasar, Manuela Oetterli, Sarah Mantwill

**Affiliations:** ^1^ Faculty of Health Sciences and Medicine University of Lucerne Lucerne Switzerland; ^2^ Swiss Learning Health System University of Lucerne, Faculty of Health Sciences and Medicine Lucerne Switzerland; ^3^ Interface Politikstudien Forschung Beratung AG Lucerne Switzerland

## Abstract

**Introduction:**

Primary care in Switzerland faces a set of interrelated systemic challenges that exemplify a wicked problem. Addressing such complexity requires participatory approaches that integrate diverse perspectives and generate context‐sensitive solutions. The project Health2040 engaged stakeholders in a co‐creation process to co‐design a future model of ambulatory primary care for Switzerland and an accompanying action plan to support its implementation.

**Methods:**

The project employed a structured co‐creation process, beginning with context exploration, evidence synthesis, and stakeholder consultations. Following the preparation phase, two co‐design workshops with more than 50 stakeholders, including patients, healthcare professionals, government authorities, social care representatives, insurers, and researchers. The workshops combined customised participatory methods such as patient personas, visioning, system mapping through building blocks, fishbowl discussions, and the X‐Curve.

**Results:**

The process produced two main outputs: (1) the ‘Health Network for All’ model, a vision for patient‐centred, network‐based, and digitally supported primary care, and (2) the Action Plan, which identifies three central action areas, profession‐specific activities, and implementation strategies. Thirty‐one individuals endorsed the plan.

**Conclusion:**

The project illustrates how co‐creation can be used to address wicked problems in health systems by linking visionary model‐building with actionable strategies. The outputs provide a framework that may inform ongoing efforts to shape the future of Swiss primary care. Beyond the Swiss context, the study illustrates how internationally established primary care principles can be operationalized through co‐creation in a local context and highlights methodological design principles that may inform similar processes in other settings.

**Patient or Public Contribution:**

Patients and members of the public contributed at different stages of the project. Stakeholder feedback informed workshop design and materials, and representatives from all relevant groups participated in developing and refining the project outputs and supporting dissemination activities. Patient representatives shared lived experiences, contributed to patient‐specific model development, and participated in group discussions during the workshops.

## 1 Introduction

Health systems are increasingly confronted with ‘wicked problems’: complex issues with unclear boundaries, contested values, and no simple solutions [1]. Originating in the work of Rittel and Webber [2], the concept distinguishes complex social policy and social science problems from ‘tame’ natural science problems that are well‐defined and solvable through linear, reductionist approaches [3]. It has since been widely adopted in public policy literature, where wickedness is understood to characterise not only social problems themselves but also the policymaking processes through which they are defined, negotiated, and addressed [4]. In health policy, wicked problems include balancing financial sustainability with health equity and access to care, responding to population aging and rising chronic conditions, or addressing workforce shortages. These challenges are all shaped by multiple behavioural, environmental, and systemic factors, involve conflicting stakeholder interests, and have no simple solutions [5, 6].

Primary care delivery has been described as a wicked problem in contexts including service provision in remote locations [7], care for people with disabilities [8], and service delivery in federated and fragmented systems [9]. In Switzerland, primary care likewise faces interrelated systemic challenges. An aging population, the rising prevalence of chronic diseases, and multimorbidity are increasing demand for continuous and coordinated care, while workforce shortages are expected to worsen, with many general practitioners approaching retirement and nurses and medical assistants leaving the profession [10, 11, 12, 13]. Cost pressures, requiring professionals to work more efficiently, further exacerbate workforce challenges while placing an increasing financial burden on the population [14]. Regional disparities in physician supply [15] and the decentralised organisation of healthcare across 26 cantons add further complexity [16], unfolding alongside broader transformations in healthcare digitalisation and emerging care models [17].

Recognising these dynamics, expert reports have long called for innovative models that ensure high‐quality, patient‐centred, and cost‐effective primary care through better care for chronically ill patients, prevention, interprofessional collaboration, and stakeholder involvement [18]. Strengthening primary care has thus become a policy priority in Switzerland, most recently reflected in the *Agenda Grundversorgung* (Primary Healthcare Agenda), which aims to promote innovative care models, professional role development, digital opportunities, and improved working conditions [17]. The agenda is being developed with broad stakeholder participation, including actors from beyond primary care, reflecting the need for intersectoral collaboration. Indeed, given the complexity of these challenges, traditional top‐down transformation strategies are unlikely to be sufficient. Addressing them requires approaches that incorporate diverse stakeholder perspectives, support knowledge‐sharing, and create spaces for deliberation of competing perspectives [4, 6].

In this context, co‐creation has been proposed as a promising strategy for developing solutions for complex social and health system challenges that reflect stakeholder needs [19, 20, 21]. It represents a collaborative approach to problem‐solving in which diverse stakeholders are engaged throughout all project phases, from problem identification to implementation [22]. By drawing on collective knowledge, lived experiences, and expertise of participants, co‐creation can foster innovative solutions that are less likely to emerge from hierarchical or top‐down processes [23, 24]. Moreover, co‐created solutions may be more acceptable to stakeholders because they have participated in their development [25, 26, 27].

Against this background, co‐creation was adopted in a project conducted within the larger research initiative *‘*Health2040’ to conceptualise responses to the wicked problems facing primary care in Switzerland. At project initiation, participatory spaces in Swiss primary care already existed, including the federal *Forum Medizinische Grundversorgung* (Forum on Primary Healthcare), which brings together federal and cantonal authorities and key primary care organisations to discuss challenges, priorities, and potential solutions [28]. However, to our knowledge, few Swiss initiatives had addressed primary care as a system‐level challenge through structured co‐creation involving patients, healthcare and social care professionals, insurers, researchers, and policy and administrative decision‐makers within the same process. The present project was distinctive in engaging these heterogeneous actors in a co‐creation process to jointly develop a future organisational model of primary care and implementation‐oriented activities. More recent developments, including the participatory development of the federal *Agenda Grundversorgung* (Primary Healthcare Agenda) and the *Bevölkerungsrat 2025 (Citizens’ Assembly 2025)*, further illustrate the growing use of stakeholder and citizen participation in Swiss health policy [17, 29].

This paper examines the resulting co‐creation process as a structured methodological approach for translating a complex and contested problem space into a coherent model of primary care and an accompanying implementation plan. Its contribution lies not only in the resulting model, but also in providing a transparent, process‐oriented account of how evidence‐informed inputs, diverse stakeholder perspectives, iterative refinement, and implementation‐oriented planning were combined in a real‐world health system context. Such transparent documentation is important given recent evidence showing considerable variation in how co‐creation is defined and applied in primary care [30]. Below we present the project's co‐creation framework, methods, and the outputs. While the resulting model is Swiss‐specific, the conceptual and methodological insights generated through this process may inform similar initiatives seeking to address complex primary care challenges through co‐creation, provided they are adapted to local health system context.

## 2 Methodology

### 2.1 Project's Co‐Creation Framework

The project aimed to engage relevant stakeholders in a structured co‐creation process with two main goals: (1) to co‐design a model representing a shared vision for the future of ambulatory primary care in Switzerland; and (2) to co‐develop an action plan for implementing the model.

To guide the process, we adapted the Generative Co‐Design Framework for Healthcare Innovation, which offers a structured approach to translating creative contributions of end‐users into healthcare innovations [31]. Developed to support healthcare innovators in redesigning processes or systems through participatory engagement, the framework emphasises iterative, user‐driven design and the translation of experiential knowledge into actionable solutions. These features made it well suited to our project, which aimed to generate innovative, stakeholder‐informed responses to challenges facing Swiss primary care.

The Health2040 Co‐Creation Framework followed three stages: (1) Pre‐Design, (2) Co‐Design, and (3) Post‐Design. Figure 1 summarises the overall process and timeline.

**Figure 1:**
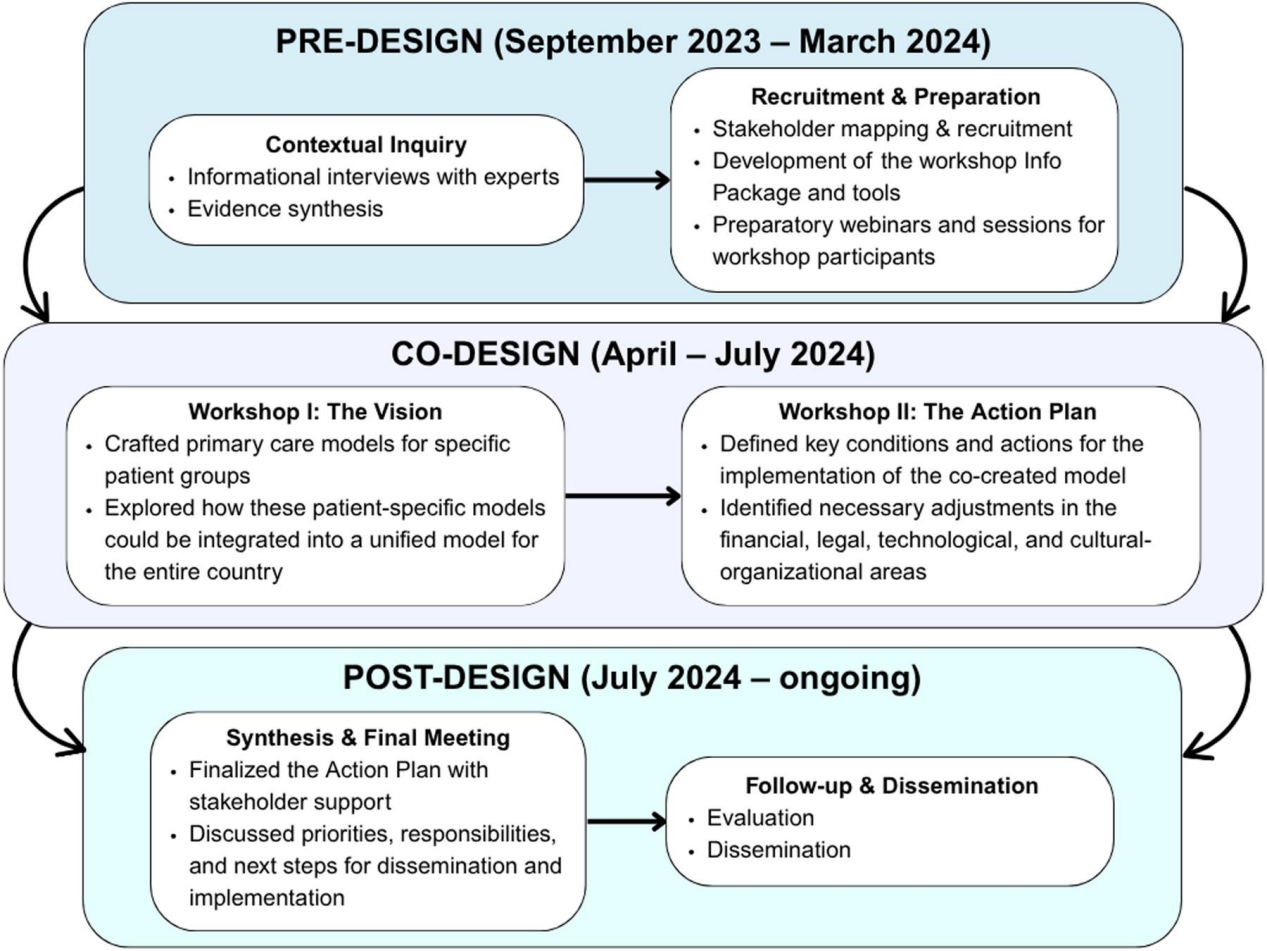
Project's co‐creation framework.

### 2.2 Stage I: Pre‐Design – Context Exploration and Preparation

During the Pre‐Design stage, we explored the challenge context through evidence synthesis and expert consultations, and prepared for co‐creation by recruiting stakeholders and developing workshop materials and tools.

#### 2.2.1 Contextual Inquiry

At the outset, a project team was formed comprising a project leader, a junior project leader, a project manager, three scientific collaborators, and a facilitator. After agreeing on a project concept involving evidence synthesis and two workshops, we conducted brief telephone interviews with six experts (‘the expert group’) with whom we had previously collaborated. The interviews gathered feedback on workshop design, preparatory materials, and topics for evidence synthesis, informing subsequent refinements.

The evidence synthesis involved a literature review of innovative primary care models in Switzerland. This step was essential to uphold core principles of co‐creation, which emphasise tailoring the process to its specific context and grounding it in research and empirical evidence to ensure that decisions are informed by scientific knowledge and best practices [32]. The analysis identified key building blocks of such models and illustrated them with concrete examples [33]. We also synthesised findings from project substudies on the preferences of the general population and healthcare professionals regarding the future design of ambulatory primary care [14, 34]. Together, these sources provided the evidence base for workshop discussions and anchored the co‐created outputs in existing evidence on stakeholder preferences and ongoing initiatives.

#### 2.2.2 Recruitment

Potential participants were initially identified based on the project team's expertise and professional networks. To reduce the risk of overlooking relevant actors, the preliminary list was reviewed against a predefined stakeholder map and discussed with the expert group, with deliberate consideration of stakeholder groups and linguistic regions. The stakeholder map (Figure 2) was based on the structure of the Swiss ambulatory primary care system, team expertise, and relevant literature [35, 36, 37, 38].

**Figure 2:**
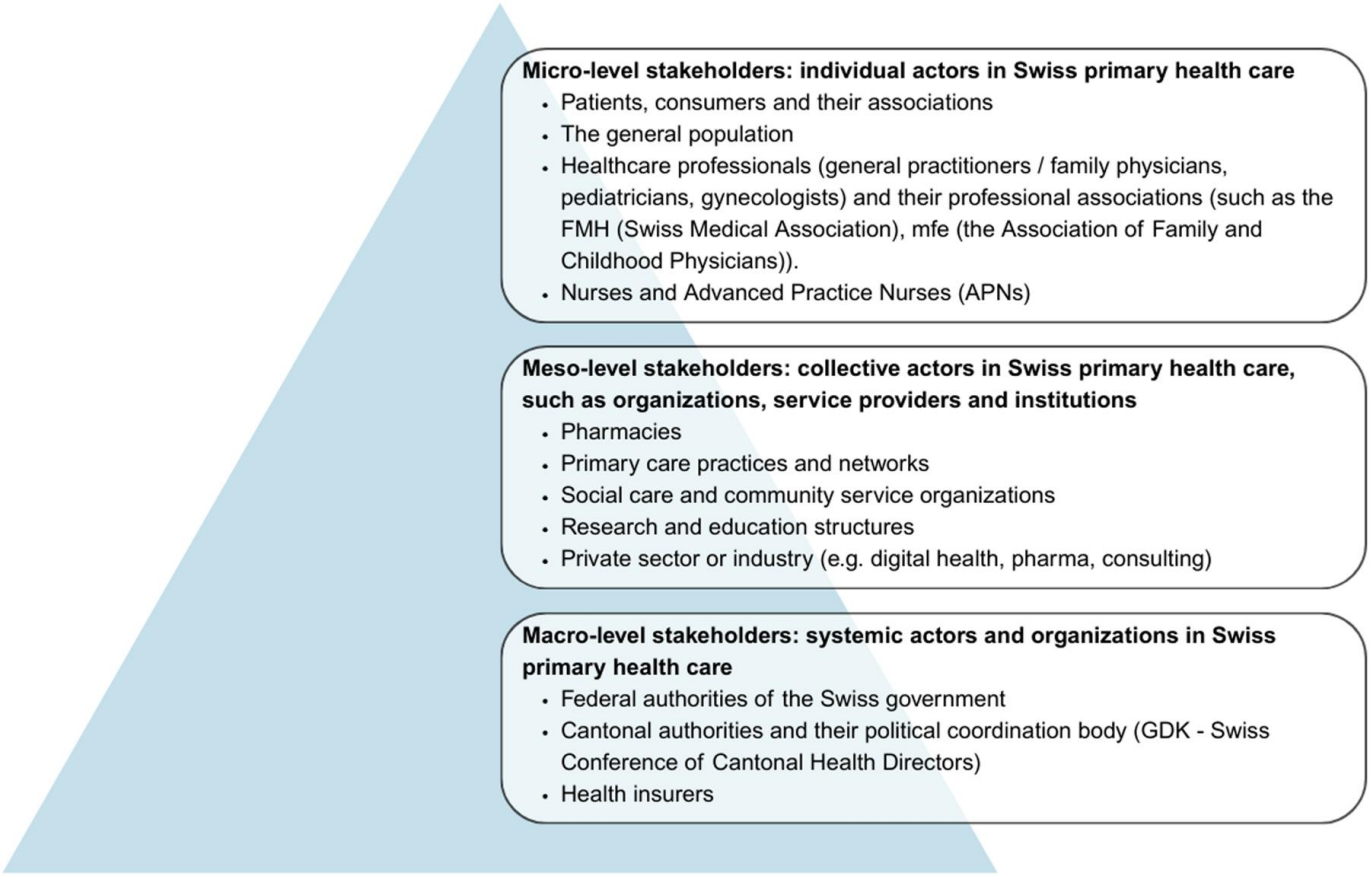
Stakeholder map of actors in Swiss primary care.

The resulting recruitment list comprised 90 potential participants. Recruitment was purposive and aimed to achieve broad contextual representation across professional and organisational backgrounds, different roles within the primary care system, and Switzerland's German‐, French‐, and Italian‐speaking regions [39]. Patient representatives were recruited purposively based on characteristics corresponding to the patient profiles used in the first workshop (*see Workshop I*) to anchor the discussion in relevant lived experience.

The recruitment target was a minimum of 30 participants at each workshop. Invitations for both workshops were sent by e‐mail 3 months before the first event, followed by a reminder 2 months before. Participation in both workshops was free of charge, with meals provided by the organisers. Patient representatives additionally received monetary compensation, including reimbursement for travel costs.

#### 2.2.3 Preparation

We developed preparatory materials (*‘Info Package’*) for the workshops, comprising (1) an evidence brief presenting findings from the literature review on innovative primary care models in Switzerland [33], (2) summaries of the project substudies [14, 34], and (3) instructions on the workshop agenda, activities, and logistics. The Info Package was refined based on expert group feedback to ensure clarity and relevance.

We also developed two workshop tools – *the Primary Care Model's Building Blocks* and *the Patient Profiles* – based on the evidence synthesis and stakeholder consultations. Three healthcare professionals provided input to ensure the patient profiles reflected real‐world patient groups, while researchers involved in earlier project substudies supported integration of prior findings.

Two weeks before the first workshop, we held preparatory webinars to familiarise participants with the workshop structure, introduce them to one another, and foster engagement. The Info Package was shared in advance. Two 1.5‐h sessions were offered (with recordings), featuring icebreakers, an overview of workshop objectives and content, and testimonials from a patient representative and a cantonal authority representative for inspiration and strengthening commitment to the co‐creation process. Separate preparatory sessions were offered to patient representatives for additional support and clarification.

### 2.3 Stage II: Co‐Design – Participatory Workshops

The Co‐Design stage represented the core of the co‐creation process, implemented through two in‐person, full‐day, multi‐stakeholder participatory workshops. These aimed to co‐develop a primary care model (Workshop I) and an accompanying action plan (Workshop II), drawing on diverse stakeholder perspectives and the pre‐design evidence synthesis. This sequential approach ensured that the project not only fostered innovative ideas and shared visions but also translated them into practical and executable strategies.

#### 2.3.1 Setting and Structure

Both workshops combined small‐group work with plenary sessions and presentations (see agendas in Supplementary File 1). They were held in unconventional venues: the first in a creative event space and the second in a former indoor swimming pool transformed into a cultural venue. These settings were chosen to break away from traditional clinical or academic environments and to foster open, imaginative engagement among participants, as literature suggests that novel, unconventional environments can drive curiosity and stimulate creativity [40, 41]. Workshops were conducted in German and Swiss‐German. To ensure skilled facilitation, we developed facilitator guides outlining the workshop programme, group activities, and facilitation prompts, and hosted training sessions for facilitators. Workshop methods were selected based on their fit with the objectives of each activity, suitability for a heterogeneous stakeholder group, feasibility within the available time and resources, and discussion within the project team. Particular attention was paid to structured facilitation and participation formats intended to create opportunities for all participants to contribute and for different perspectives to be expressed.

#### 2.3.2 Data Collection and Analysis

Group discussions were audio‐recorded, transcribed, and documented through flipcharts, facilitator notes, and discussion protocols. These materials were reviewed and discussed within the project team to identify common themes and points of consensus across groups, informing post‐workshop outputs that were subsequently shared with participants for review.

#### 2.3.3 Workshop I (April 2024)

The first workshop aimed to co‐create a shared vision for future ambulatory primary care for Switzerland, represented as a primary care model. It began with an agenda overview, introductions, and an icebreaker, ‘Tell your Story by Means of an Object’ [42], in which participants shared an object symbolising their connection to primary care. This activity was intended to create an open atmosphere and encourage reflection on participants’ experiences through symbolic representation.

Participants were then assigned to five mixed‐stakeholder groups to ensure diversity of perspectives, a factor shown to enhance collaborative problem‐solving [43]. Each group was facilitated by a project team member, with support from researchers involved in earlier project substudies.

The first group work session focused on developing patient‐specific primary care models. Each group was assigned one of five Patient Profiles created using the *Personas* method [39] and informed by the evidence synthesis: (1) a young adult with mental health challenges; (2) an older person with multiple chronic conditions living in a rural area; (3) a middle‐aged working adult facing an acute health episode; (4) a parent of a child with behavioural problems; and (5) a person with a migration background and limited proficiency in local languages. The profiles, combining textual descriptions and images, served to build a shared understanding of service users and to ground the model in their needs (see Supplementary File 2). To further anchor discussions in lived experience, patient representatives with characteristics similar to the patient profiles opened the discussions by sharing two positive and two negative experiences with primary care.

Using twelve predefined Building Blocks (e.g., location, scope, services, providers, digital technologies; see Supplementary File 2), groups selected, adapted, and combined different components to design models. Each building block could be specified through one or more predefined options (e.g., local, regional, or national scope), with blank option cards and a ‘joker’ building block for additional ideas. Methodologically, the exercise combined *visioning* – supporting stakeholders in articulating a shared vision and concrete ideas for future care [44] – with *systems mapping*, used to represent interdependencies among system elements [45]. The building blocks operationalized these approaches by offering a structured but adaptable way to visualise and compare possible models.

Groups documented discussions on flipcharts, assembled selected building blocks on A0 posters to visualise their models, and prepared short presentations highlighting three key considerations behind their design for cross‐group exchange in plenary.

The second group work session focused on synthesising the five patient‐specific models. Participants were reorganised into five new groups, each including members who had worked with different patient profiles in the previous session to represent all patient perspectives.

Groups first identified commonalities and differences across models and defined shared core elements, then clustered remaining components into patient‐specific branches, highlighting particular needs (e.g., elderly and migrant populations; regional vs. national scope). Finally, they discussed implementation requirements and identified three priority *Action Areas* (*Entwicklungsfelder*, e.g., education, reimbursement, professional networking, data exchange, legal frameworks, organisational change). Results were documented on A0 posters and presented in short plenary pitches.

Following the workshop, the project team analysed discussion protocols, transcripts, notes, and the group work outputs to develop a draft synthesised model and list of Action Areas, which were discussed with participants and shared in advance as inputs for Workshop II.

#### 2.3.4 Workshop II (June 2024)

Workshop II shifted from visioning to action planning, with participants defining *framework conditions* (Rahmenbedingungen) and *activities* (Massnahmen) for model implementation.

It began with a plenary recap of Workshop I, presenting the synthesised model and three Action Areas. Short video interviews with Swiss projects and organisations illustrated how similar ideas were already being applied in practice, drawing on experience‐based co‐design principles, which emphasise the power of narratives (in this case, implementation stories) to inspire practical ideas for service improvement [46]. Throughout the day, professional graphic recording visually documented discussions in real time.

In the first group work session, participants discussed *framework conditions* for implementing the primary care model, focusing on financial, legal, technological, cultural‐organisational, and personnel domains. Four mixed‐stakeholder groups (~ 10 participants each) used the fishbowl method across three 30‐min rounds, rotating participants between inner ‘discussion’ and outer ‘listening’ circles to create opportunities for different participants to enter the discussion [44]. Group work canvases (Supplementary File 2) were structured using the X‐Curve [47] to reflect on systemic change and transition toward the co‐created model, identifying practices to improve, abolish, introduce, or institutionalise within each Action Area. Inputs were documented on X‐Curve posters by facilitators and note‐takers. Participants then reflected individually on framework conditions most central to their professional roles and the changes required.

The second group work session focused on profession‐specific *activities* addressing the framework conditions. Participants were grouped by professional background (e.g., health professionals, patients, education, research, social professions; seven groups). Working in relatively homogeneous groups aimed to enable participants to draw on shared expertise and identify feasible, profession‐specific actions, complementing the mixed‐stakeholder discussions in the preceding session [48]. Participants first shared their individual reflections, after which groups prioritised key conditions, documented them in structured templates (Supplementary File 2), and defined concrete activities their profession could initiate. Groups then visited each other's posters in a gallery walk and added feedback. Finally, each group presented three priority activities in plenary, which were discussed and rated using Mentimeter (an interactive online tool for real‐time polling and feedback). The workshop concluded with next steps for consolidating activities into an Action Plan and developing a dissemination strategy.

### 2.4 Stage III: Post‐Design – Refinement and Dissemination

The Post‐Design stage focused on finalising the Action Plan and initiating dissemination.

After Workshop II, the project team consolidated outputs into a draft Action Plan outlining the model, Action Areas, and implementation activities. Participants reviewed the draft through a survey and document comments, providing feedback on clarity, completeness, missing aspects, implementation challenges, willingness to support the plan, potential roles in implementation and dissemination, and priority success factors for the Action Areas.

Based on this feedback, the Action Plan was revised and discussed in a final online meeting in September 2024 to clarify priorities, responsibilities, timelines, and next steps. The finalised Action Plan was launched in November 2024 on the project website.

#### 2.4.1 Dissemination Activities

Following the Action Plan launch, dissemination efforts have focused on raising awareness and supporting diffusion into practice and policy. Dissemination is integral to research, as it enhances the visibility and understanding of results among stakeholders and supports implementation and policy uptake [49]. In line with the co‐creation approach, participants and their organisations were encouraged to actively communicate the model and Action Plan within their networks. Initial activities in 2024 included a media release, promotion via social media channels and newsletters, and publications on stakeholder organisations’ websites. The Action Plan was also presented to key policy bodies, professional associations, and governmental authorities.

Dissemination continues through presentations at organisational events, industry forums, and academic conferences, as well as through lectures, scientific publications, and podcasts [50]. A private LinkedIn group supports ongoing exchange around follow‐up activities.

#### 2.4.2 Evaluation

The project's evaluation strategy covered participants’ expectations, satisfaction with the workshops, engagement quality, acceptance of the outputs, and reflections on experiences with the co‐creation process, including perceived strengths and challenges associated with the different methods used. Data were collected through pre‐ and post‐workshop surveys, semi‐structured interviews with participants, and a focus group with project team members. Results of this evaluation will be reported in a separate publication.

## 3 Results

### 3.1 Stakeholder Participation

A total of 55 unique stakeholders participated in at least one stage of the process. The first workshop brought together 38 participants, while 33 attended the second, and 17 joined the final online meeting. Of the 38 participants in Workshop I, 16 also attended Workshop II (42%), and eight participated in all three events (21%). Participation was flexible: participants were not expected to attend every activity and could provide feedback after each stage as an alternative contribution.

Across the overall process, participants represented a wide spectrum of stakeholder groups, including patients, healthcare professionals, political actors, social care organisations, education and research, health insurers, healthcare providers, and other key actors (Table 1). Among them, 40% held leadership or senior management roles (e.g., policymakers, organisational heads), 20% advisory or operational roles (e.g., researchers, mid‐level managers), 20% were frontline professionals (e.g., physicians, nurses, social workers), and 20% represented the community or user level (e.g., patients or community representatives).

**Table 1:** Distribution of stakeholder groups across stages of the co‐creation process.

Stakeholder group	Overall (*N* = 55), n (%)	Workshop I (N = 38), n (%)	Workshop II (N = 33), n (%)	Final meeting (N = 17), n (%)
Healthcare professionals, health service providers and networks	14 (25%)	10 (26%)	10 (30%)	6 (35%)
Social care providers and organisations	12 (22%)	7 (18%)	7 (21%)	4 (24%)
Patients and patient organisations	11 (20%)	10 (26%)	2 (6%)	1 (6%)
Private sector (incl. pharma, think tanks and consulting)	6 (11%)	4 (11%)	5 (15%)	3 (18%)
Education structures and research	5 (9%)	3 (8%)	5 (15%)	1 (6%)
Government	4 (7%)	3 (8%)	1 (3%)	1 (6%)
Pharmacies	2 (4%)	0 (0%)	2 (6%)	0 (0%)
Health insurers	1 (2%)	1 (3%)	1 (3%)	1 (6%)
**Total**	**55 (100%)**	**38 (100%)**	**33 (100%)**	**17 (100%)**

Stakeholder representation varied across stages. The largest decline was observed among patients, from 10 participants in Workshop I (26%) to two in Workshop II (6%) and one in the final meeting (6%). In contrast, healthcare providers and social care organisations remained comparatively well represented across stages.

### 3.2 Outputs of the Co‐Creation Process

The process produced two core outputs: (1) the ‘Health Network for All’, a conceptual model for future Swiss primary care, and (2) an Action Plan outlining implementation activities. They represent a synthesis of empirical evidence and stakeholder perspectives, developed through the co‐creation process. Supplementary File 3 provides examples of intermediate workshop outputs and illustrates how these informed the final model and Action Plan.

#### 3.2.1 The ‘Health Network for All’ Model

##### 3.2.1.1 Conceptual Overview

The model ‘Health Network for All’ («Gesundheitsnetz für alle – pour tous – per tutti») represents a concept for a future ambulatory primary care system in Switzerland (see Figure 3). It conceptualises primary care as a complex, interconnected system that integrates medical, social, and community dimensions. Grounded in the biopsychosocial perspective, the model defines primary care as network of actors and settings that collectively respond to population needs. It is organised around six defining characteristics:

1. Population orientation: The model addresses the whole population, while recognising the specific needs of major patient groups (based on the patient profiles).
2. Patient participation and empowerment: Patients are actively involved in decisions concerning their treatment and supported in strengthening health literacy. They can choose how to access network services according to their preferences and needs.
3. Integration across sectors: Healthcare providers, social services, community organisations, and civil society actors are part of the model. They collaborate in a highly interprofessional, interdisciplinary, and data‐driven way, built on mutual trust.
4. Network‐based organisation: The model follows a digitally supported, network‐based design. Each network serves around 15,000 people, with the exact form of local and regional networks adapted to the local context, population needs, professional preferences, geography, and existing structures.
5. System‐level collaboration: Individual networks collaborate on a higher‐level platform to share knowledge, use synergies, and define interfaces with inpatient care.
6. Health promotion and prevention: Networks incentivize health promotion and prevention, reframing primary care from a disease‐centred to a person‐centred and health‐oriented paradigm.

**Figure 3:**
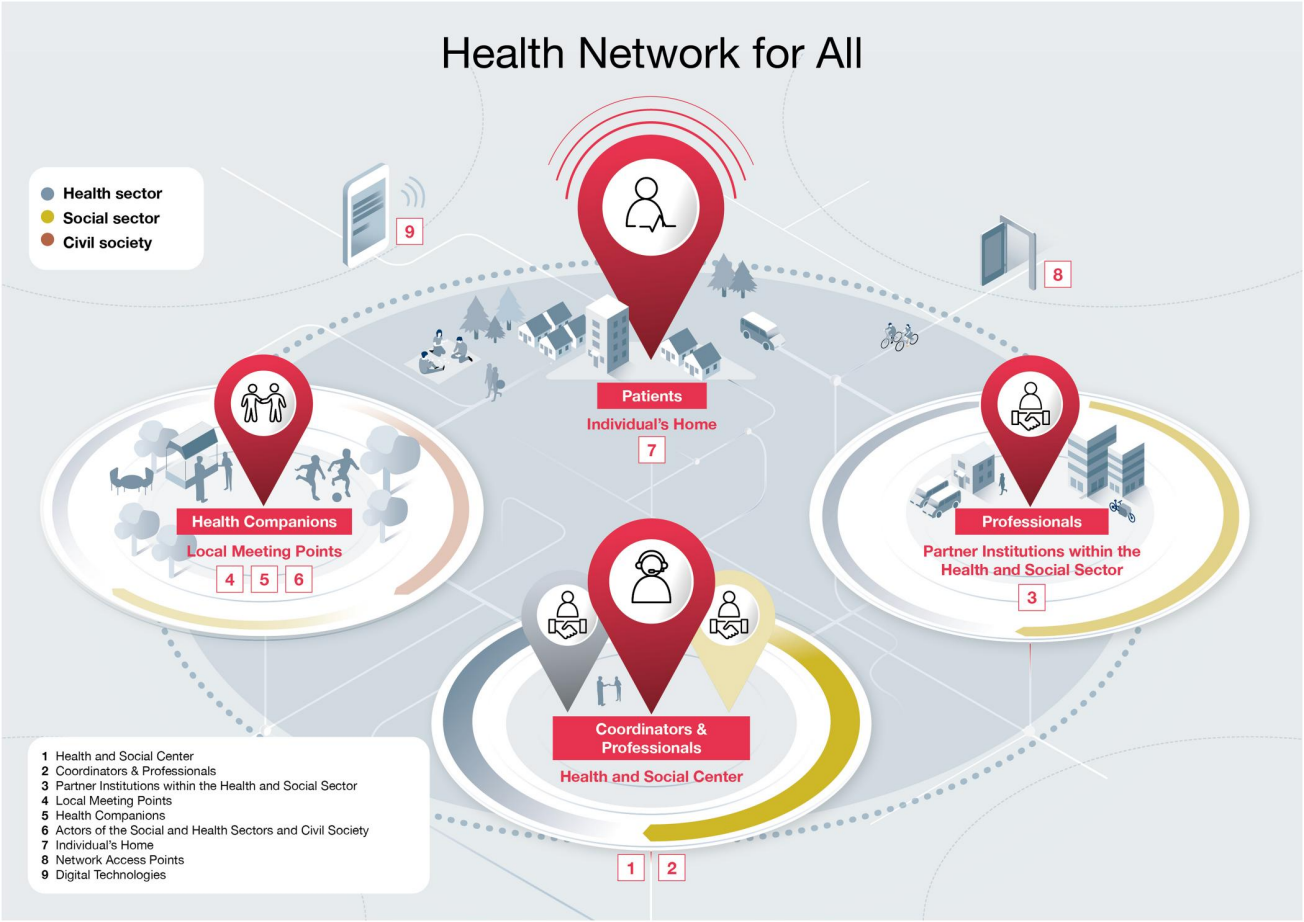
The ‘Health Network for All’ Model.

Together, these characteristics position the model as a learning and adaptive system to local needs, while also balancing it with national priorities.

##### 3.2.1.2 Structural Elements of the Model

The co‐created model can be described through nine central elements (see Figure 3):

1. *Health and Social Centre*
  At the core of each network is a *Health and Social Centre* that unites medical, therapeutic, and social services under one roof (e.g., physicians, therapists, counselling professionals, nursing staff, advanced practice nurses, social workers, pharmacists). Mobile teams may also be affiliated and can extend care into the community and patients’ homes. The centre delivers health promotion, prevention, curative, rehabilitative, and palliative care through close interprofessional collaboration.
2. *Coordination Staff*
  Specialised *Coordination Staff* who triage patient requests and act as central contact points at the *Health and Social Centre* for patients and providers. For complex cases, they coordinate services within and beyond the centre, connecting patients with local meeting points, partner institutions, or actors from social services and civil society.
3. *Partner Institutions*
  The network collaborates with *Partner Institutions*, such as hospitals, nursing homes, specialists, therapists, and community counselling or support services located within the network's catchment area. These partnerships are formalised through established collaboration structures and referral pathways that enable reciprocal patient transfers.
4. *Local Meeting Points*
  Embedded in the community, *Local Meeting Points* are easily accessible spaces that connect residents with social and health services and civil society. They provide psychosocial support for patients and families and can serve as informal entry points into the health network. Examples include neighbourhood centres, youth clubs, or school‐based social work programmes. Meeting points may host social and cultural activities (e.g., cafés, workshops, youth programmes) and are supported by *Health Companions* who help especially young people, older adults, migrants, and caregivers to access care.
5. *Health Companions*
*Health Companions*, often volunteers with diverse backgrounds, serve as low‐threshold contacts at local meeting points. They listen to concerns, provide information, promote health literacy, and connect individuals with appropriate services, referring them to the *Health and Social Centre* or *Coordination Staff* when needed. Training and professional support are provided when needed.
6. *Actors of the Social and Health Sector and Civil Society*
  Neighbourhood groups, youth services, leisure associations, and support organisations collaborate with *Local Meeting Points* to complement medical care, providing psychosocial support while promoting health literacy, prevention, and health promotion. *Health Companions* help residents connect with these services.
7. *Individual's Home as a Care Setting*
  The home remains a key care setting. Depending on needs, patients receive medical, nursing, household, and psychosocial support in their homes, delivered by mobile teams, partner institutions, family, and community actors.
8. *Network Access Points*
  Access to the network is flexible and multimodal. Individuals may contact the network through *Local Meeting Points*, scheduled visits to the *Health and Social Centre*, *Partner Institutions* or digitally via an app.
9. *Digital Technologies*
  A unified *Digital Platform* ensures secure data exchange across the network. Both providers and patients can access their data, supporting coordinated care. Artificial intelligence (AI)‐based tools assist with coordination and decision‐making, while an app provides appointment booking and information on services and general health topics in multiple languages.

#### 3.2.2 The Action Plan

The Action Plan provides strategic directions for implementing the ‘Health Network for All’ by 2040. It specifies three key *Action Areas*, their success factors (understood as conditions important for advancing each area, rather than formal monitoring indicators), concrete implementation activities, and potential initiating actors. The *Action Areas* are:

1. *Network‐Based Care* focuses on strengthening collaboration across health and social sectors and civil society to ensure flexible, person‐centred care. Success depends on the broad engagement of professionals, citizens, and policymakers, sustainable financing that recognises coordination work, trust‐building through interprofessional education, and legal or policy frameworks that formalise network‐based provision.
2. *Coordination and Navigation* addresses the alignment of services and patient guidance through the network, supported by a shared data platform. Key success factors include establishing and defining coordination roles, enabling secure information exchange, and providing support to patients navigating care pathways.
3. *Low‐Threshold and Flexible Access* aims to enable rapid and equitable entry to the network. Access routes include multilingual apps and local community spaces that ensure that everyone can easily access services within their living environment. Success factors include promoting health literacy, supporting civil society engagement and volunteer wo**r**k, and ensuring transparent, up‐to‐date service information.

The full Action Plan is available on the project website [51] and has been endorsed by 31 individuals. Since its publication, the model and Action Plan have contributed to subsequent policy and implementation initiatives at different levels of the health system. Their inclusion as an input to the national Primary Healthcare Agenda in 2025 [52] indicates that the co‐created outputs reached national policy processes, informing ongoing efforts to shape primary care in Switzerland. At the municipal level, the initiative *Gesundheitswegweiser Luzern* (‘Health Guide Lucerne’) launched in 2026 under the cantonal integrated care strategy (IGeL), implements the vision laid out by the Action Plan, aiming to establish a regional integrated care network with a central coordination office [53, 54]. Additionally, the project team used the model and Action Plan to support or advise other projects at regional and local levels, including integrated care initiatives in Lucerne and Graubünden.

## 4 Discussion

This discussion considers both the value and challenges of applying co‐creation in a complex, multi‐stakeholder health system setting. We first consider its contribution to addressing wicked problems and advancing understandings of primary care, before reflecting on methodological lessons, challenges, and limitations.

### 4.1 Co‐Creation As a Response to Wicked Problems in Health Systems

Swiss primary care faces systemic challenges that constitute a wicked problem, requiring transformation beyond top‐down reforms and approaches that integrate diverse perspectives and generate context‐sensitive solutions. The Health2040 project sought to address this need through a structured, evidence‐informed co‐creation process. More than 50 stakeholders jointly developed two key outputs: the ‘Health Network for All’ model, a vision for patient‐centred, network‐based, and digitally supported primary care, and the Action Plan, which outlines Action Areas, profession‐specific activities, and strategies for implementation.

The process illustrated how co‐creation can help generate practical responses to wicked problems in health systems. By linking visionary model‐building to action planning, the project addressed two central challenges of wicked problems: creating shared understanding across heterogeneous groups and moving from ideas to action [55]. The workshops showed how structured and tailored co‐creation methods and tools can support participants in navigating transition dynamics, reconciling different perspectives, and identifying opportunities for systemic change. The resulting model and Action Plan are not definitive solutions but adaptive strategies to guide experimentation and collaboration across sectors. Endorsement by 31 individuals indicates support for the proposed direction.

### 4.2 Advancing Conceptual Understandings of Primary Care

At a conceptual level, the ‘Health Network for All’ model advances understandings of primary care by conceptualising it as an integrated network linking medical, social, and community resources into a new organisational model [56]. Its design operationalizes the five core functions of primary care: (1) *first‐contact accessibility* through local meeting points, health companions, and diverse entry routes; (2) *continuity* via coordination staff and a shared data platform; (3) *coordination* through interprofessional collaboration, partnerships, and secure data exchange; (4) *comprehensiveness* by integrating promotive, preventive, curative, rehabilitative, and palliative services at Health and Social Centres; and (5) *person‐centeredness* through shared decision‐making and flexible care pathways that respect individual preferences [57, 58]. The model also emphasises community orientation – another foundational element of primary care – through community spaces as entry points, health companions as trusted contacts, and community services complementing medical care, reflecting a bio‐psycho‐social approach [57].

Notably, the core functions of primary care were not introduced to participants *a priori*, yet the co‐created model closely reflected these established principles. This suggests that co‐creation can do more than structure stakeholder input: it can surface implicit values and shared conceptual understandings that underpin complex systems such as primary care.

The Action Plan translates these principles into implementation priorities, showing how they can operate together as a coherent, interdependent system. This convergence with internationally established primary care functions suggests that, although the resulting model is context‐specific, it reflects principles that extend beyond Switzerland. By operationalizing these functions through concrete organisational structures, roles, access routes, and implementation activities, the project connects the locally developed model to the broader international evidence base on effective primary care. This may offer useful insights for other settings seeking to adapt the same principles to their local context.

### 4.3 Methodological Reflection: Lessons from Co‐Creation

The process yielded several methodological insights into how co‐creation can be applied in complex, multi‐stakeholder health system settings.

First, the process underscored the importance of grounding co‐creation in evidence‐informed inputs (i.e., the model's Building Blocks, the Patient Personas, and the Evidence Brief) to anchor creativity and lived experiences in existing knowledge. This illustrated how co‐creation can serve as an additive approach that combines research evidence with experiential expertise [23]. Patients, healthcare and social care professionals, researchers, and policy and administrative actors contributed perspectives from different levels of the primary care system. Bringing these perspectives together supported the development of a model that extended beyond medical service delivery to incorporate coordination, social support, and system‐level implementation requirements, linking the perspectives of those experiencing and delivering care with those involved in organisational and policy decision‐making.

Second, the participatory methods supported dialogue among heterogeneous actors and the development of tangible outputs. Several design elements likely contributed to this. Facilitation by topic experts who received dedicated training was intended to ensure productive discussion, consistent with prior research suggesting that skilled facilitation helps participants engage and navigate complex discussions [59]. User‐friendly, visually structured tools aimed to lower barriers across diverse professional backgrounds, consistent with findings that co‐creation tools are most beneficial when they are easy to use and require little prior expertise [59]. By helping to visualise complex systems and relationships, the methods encouraged participants to focus on broader feedback loops rather than isolated details, supporting higher‐level problem‐solving [59]. Iterative feedback mechanisms allowed outputs to be refined across project stages and participants to see how their contributions were carried forward. Evidence suggests that continuous stakeholder involvement across the entire co‐creation process can foster innovation, stronger partnerships, and commitment to use the knowledge produced [60, 61]. A separate publication reporting the process evaluation examines these design components in greater depth, including participant experiences and practical challenges associated with the workshop methods.

Lastly, our project highlights the importance of transparent process documentation. While co‐creation is increasingly used, many studies lack detailed reporting on processes, method selection, and adaptation, creating barriers to understanding and replicating such initiatives [23, 62, 63]. Our study addressed this gap by providing a process‐oriented account of method development and application in a real‐world setting. Such documentation can also support accountability by making stakeholder participation, methodological choices, and the progression from stakeholder input to final outputs more traceable. Although specific methods need to be adapted to local contexts, the underlying design principles may inform similar co‐creation processes in other settings. These include combining evidence‐informed inputs, heterogeneous stakeholder involvement, structured participatory methods, iterative refinement, and implementation‐oriented action planning. In this way, the study contributes to the evidence base for participatory approaches in health system innovation.

### 4.4 Challenges and Limitations

While the project successfully produced outputs, several challenges remain. Recruitment targets were met, with participation in both workshops exceeding 30 people, yet continuity of participation across stages was moderate, reflecting the difficulty of sustaining engagement in a time‐intensive, multi‐stage process. The decline was particularly pronounced among patients and patient organisations. Patient representatives contributed substantially to Workshop I, where lived‐experience perspectives informed patient‐specific models, and outputs from this stage were carried forward. However, their lower direct participation in later stages patient input into further refinement of the outputs. Sustained engagement is a well‐documented challenge in time‐intensive co‐creation processes [59]. In retrospect, more deliberate planning for sustained patient engagement, including flexible participation options and targeted follow‐up between activities, may have strengthened continuity [64].

Stakeholder representativeness presented another challenge, common in co‐creation initiatives where small sample sizes and self‐selection can limit inclusivity and generalisability [59]. While the workshops engaged diverse stakeholders, participation from French‐ and Italian‐speaking Switzerland was limited. Age, socioeconomic status, and race or ethnicity were not used as recruitment criteria or systematically collected, limiting assessment of representativeness on these dimensions. Purposive recruitment and voluntary participation may have also introduced selection and self‐selection bias. To partly mitigate these limitations, workshop inputs drew on nationally representative surveys of preferences for future primary care across the Swiss population. Additionally, recruitment was guided by a stakeholder map and consultation with the expert group to broaden participant selection beyond the project team's immediate networks.

Representation also relates to power dynamics. The relatively high proportion of senior decision‐makers may have strengthened the implementation relevance of the process by involving actors with organisational or policy influence, while frontline and user‐level participation brought practice‐based and lived‐experience perspectives. However, representation does not imply equal influence [65]. Although facilitation and participation formats aimed to create opportunities for all participants to contribute, no explicit mechanisms systematically assessed or balanced differences in influence, and we cannot determine whether some perspectives shaped the outputs more strongly than others. As Roura [66] notes, participatory techniques can support dialogue but do not eliminate hierarchies or differences in influence. Future co‐creation processes should incorporate more explicit mechanisms to reflect on and address such dynamics.

Implementation poses another challenge. Translating the model and Action Plan into practice requires alignment across financial, legal, technological, cultural‐organisational, and personnel domains and coordinated action at federal, cantonal, and local levels. Although the Action Plan received stakeholder endorsement and participants identified possible implementation roles, these did not amount to formal commitments. However, the project aimed to develop outputs that could inform and inspire subsequent action rather than implement or monitor individual activities. Early examples of uptake described above indicate that the model and Action Plan have already informed several policy and implementation initiatives. Their further impact will depend on whether relevant actors take forward proposed activities, as limited follow‐through often constrains the real‐world impact of co‐creation [59]. Ongoing dissemination is therefore important for supporting uptake.

Methodological challenges also emerged. Co‐creation lacks standardised frameworks for method selection and adaptation [59]. Our design choices were context‐driven, reflecting project goals and stakeholder needs, which improved contextual fit but limited direct replicability. Research stresses that co‐creation should be tailored to specific settings and informed by context analysis, as no single approach suits all contexts [63, 67]. While we documented the process through protocols, facilitator notes, and audiovisual material, independent observation or standardised evaluation tools could have further strengthened rigour but would require substantial resources.

Taken together, these challenges highlight that the contribution of co‐creation to addressing wicked health‐system problems depends not only on generating outputs, but also on sustaining engagement, securing broad representation, ensuring methodological rigour, and most importantly, translating ideas into policy and practice.

## 5 Conclusions

By combining evidence‐informed inputs with participatory methods, the project brought together diverse stakeholders to rethink how primary care can be organised. While challenges remain, particularly around sustaining engagement, ensuring broad representation, and translating outputs into practice, the process generated strategies that can guide systemic change. The ‘Health Network for All’ model and the Action Plan now provide a practical reference for shaping primary care in Switzerland. Beyond their practical relevance, these outputs contribute conceptually by illustrating how primary care can be understood as an adaptive system that embeds its core functions – accessibility, continuity, coordination, comprehensiveness, and person‐centeredness – within an integrated care model design.

## Author Contributions

**Anna Romanova:** conceptualisation, data curation, formal analysis, investigation, methodology, visualisation, writing – original draft, writing – review and editing. **Andreas Balthasar:** conceptualisation, formal analysis, funding acquisition, investigation, methodology, project administration, writing – review and editing. **Manuela Oetterli:** conceptualisation, investigation, methodology, project administration, writing – review and editing. **Sarah Mantwill:** conceptualisation, funding acquisition, investigation, methodology, project administration, supervision, writing – review and editing.

## Ethics Statement

An ethics review was requested from the Ethics Committee Northwest and Central Switzerland. The committee determined that authorisation was not required, as the project did not qualify as research involving human diseases or the structure and function of the human body.

## Conflicts of Interest

The authors declare no conflicts of interest.

## Declaration of Generative AI and AI‐Assisted Technologies in the Writing Process

During the preparation of this work the authors used ChatGPT (OpenAI, version 5.1) to improve the readability and language of the manuscript. After using this tool, the authors reviewed and edited the content as needed and take full responsibility for the content of the published article.

## Supporting information


Supporting File 1

Supporting File 2

Supporting File 3

## Data Availability

The data that supports the findings of this study are available in the supplementary material of this article. Additionally, it is openly available on the project website at https://www.health2040.ch/.

## References

[1] H. Raisio , A. Puustinen , and P. Vartiainen , “The Concept of Wicked Problems: Improving the Understanding of Managing Problem Wickedness in Health and Social Care.” Management of Wicked Problems in Health and Social Care (Routledge, 2018).

[2] H. W. J. Rittel and M. M. Webber , “Dilemmas in a General Theory of Planning,” Policy Sciences 4, (1973): 155–169, 10.1007/BF01405730.

[3] J. Lönngren and K. van Poeck , “Wicked Problems: A Mapping Review of the Literature,” International Journal of Sustainable Development & World Ecology 28, no. 6 (2021): 481–502, 10.1080/13504509.2020.1859415.

[4] B. W. Head , “Forty Years of Wicked Problems Literature: Forging Closer Links to Policy Studies,” Policy and Society 38, no. 2 (June 2019): 180–197, 10.1080/14494035.2018.1488797.

[5] J. A. Johnson and S. Ahluwalia , “Recruiting and Retaining Doctors—A Wicked Problem Needing Complex Solutions,” Postgraduate Medical Journal 101, no. 1201 (November 2025): 1229–1233, 10.1093/postmj/qgaf058. 40287833

[6] I. Kickbusch and D. Gleicher , “Governance for Health in the 21st Century"[Internet] (World Health Organization. Regional Office for Europe, 2012), https://iris.who.int/items/4040d940-3058-4674-99f3-e534cf9ad810.

[7] L. A. Caffery , O. T. Muurlink , and A. W. Taylor‐Robinson , “Social Innovation in Health Response: A Rare Case of Social Enterprise Meeting Rural Health Needs.” Regional Businesses in a Changing Global Economy (Routledge, 2022).

[8] T. Iacono , “The Wicked Problem of Delivering Primary Health Care to People With Intellectual Disabilities. Commentary on “Attitudes and Experiences of General Practitioners Who Provided Health Care for People With Intellectual Disabilities: A South Australian Perspective” (Smith & Laurence, 2021),” Research and Practice in Intellectual and Developmental Disabilities 8, no. 1 (2021): 37–45, 10.1080/23297018.2021.1910987.

[9] C. Hope and M. Taylor , “Overcoming Federation and Fragmentation Through the Development of a Regional Intersectoral, Interorganisational, Community Activated Collaborative Model – An Australian Example,” International Journal of Integrated Care 25, no. 2 (2025): 80, 10.5334/ijic.ICIC24038.

[10] Bundesamt für Gesundheit (BAG) , “Medizinische Grundversorgung und Fachkräftemangel. Faktenblatt” [Internet] (2024), https://backend.bag.admin.ch/fileservice/sdweb-docs-prod-bagadminch-files/files/2025/03/18/89650d31-32bb-411b-bef9-2dc6f8a57468.pdf.

[11] T. Carron , F. Domeisen Benedetti , A. Fringer , K. Fierz , and I. Peytremann‐Bridevaux , “Integrated Care Models in Swiss Primary Care: An Embedded Multiple Case Study,” Journal of Evaluation in Clinical Practice 29, no. 6 (2023): 1025–1038, 10.1111/jep.13891. 37427549

[12] L. Burla , M. Widmer , and C. Zeltner, "Zukünftiger Bestand und Bedarf an Fachärztinnen und ‐ärzten in der Schweiz. Teil 1: Total der Fachgebiete, Hausarztmedizin, Pädiatrie, Psychiatrie und Psychotherapie sowie Orthopädie. Schlussbericht des Obsan und des Gremiums «Koordination der ärztlichen Weiterbildung» im Auftrag des Dialogs Nationale Gesundheitspolitik (Obsan Bericht 04/2022)" [Internet]. Neuchâtel: Schweizerisches Gesundheitsobservatorium (Obsan) (2022), https://www.obsan.admin.ch/de/publikationen/2022-zukuenftiger-bestand-und-bedarf-fachaerztinnen-und-aerzten-der-schweiz.

[13] M. Lobsiger and D. Liechti , “Berufsaustritte Und Bestand Von Gesundheitspersonal in der Schweiz. Eine Analyse Auf Basis Der Strukturerhebungen 2016–2018 (Obsan Bericht 01/2021),” [Internet] Neuchâtel: Schweizerisches Gesundheitsobservatorium (Obsan) (2021), https://www.obsan.admin.ch/de/publikationen/2021-berufsaustritte-und-bestand-von-gesundheitspersonal-der-schweiz.

[14] S. K. Brandt , S. Essig , and A. Balthasar "Zukünftige ambulante Grundversorgung: Einstellungen und Präferenzen von Medizinal‐ und Gesundheitsfachpersonen ausgewählter Berufsgruppen (Obsan Bericht 06/2023)" [Internet]. Neuchâtel: Schweizerisches Gesundheitsobervatorium (Obsan) (2023), https://www.obsan.admin.ch/de/publikationen/2023-zukuenftige-ambulante-grundversorgung-einstellungen-und-praeferenzen-von.

[15] Bundesamt für Statistik (BFS) . "Arztpraxen und medizinische Grundversorgung in der Schweiz, 2018‐2021". [Internet] (2023), https://www.bfs.admin.ch/asset/de/28285298.

[16] OECD/WHO . OECD Reviews of Health Systems: Switzerland 2006 [Internet]. Paris: OECD Publishing; 2006 Oct. (OECD Reviews of Health Systems), https://www.oecd.org/en/publications/oecd-reviews-of-health-systems-switzerland-2006_9789264025837-en.html.

[17] Bundesamt für Gesundheit (BAG) [Internet]. 2025. Agenda Grundversorgung, https://www.bag.admin.ch/de/agenda-grundversorgung.

[18] Die Konferenz der kantonalen Gesundheitsdirektorinnen und ‐direktoren (GDK) und Bundesamt für Gesundheit (BAG ), “Neue Versorgungsmodelle für die medizinische Grundversorgung. Bericht der Arbeitsgruppe „Neue Versorgungsmodelle für die medizinische Grundversorgung“ von GDK und BAG"[Internet] (Schweizerische Gesundheitsdirektorenkonferenz und Bundesamt für Gesundheit, 2012), https://www.gdk-cds.ch/fileadmin/docs/public/gdk/dokumentation/berichte/Neue_Versorgungsmodelle_fuer_die_medizinische_Grundversorgung.pdf.

[19] D. Heimburg and V. Cluley , “Advancing Complexity‐Informed Health Promotion: A Scoping Review to Link Health Promotion and Co‐Creation,” Health Promotion International 36, no. 2 (April 2021): 581–600, 10.1093/heapro/daaa063. 32810227

[20] G. R. Longworth , D. M. Agnello , S. Chastin , et al., “Evaluating the Co‐Creation Process in Public Health Interventions: The Proseco Framework,” Public Health 245 (August 2025): 105783, 10.1016/j.puhe.2025.105783. 40449476

[21] A. F. G. van Woezik , L. M. A. Braakman‐Jansen , O. Kulyk , L. Siemons , and J. E. W. C. van Gemert‐Pijnen , “Tackling Wicked Problems in Infection Prevention and Control: A Guideline for Co‐Creation With Stakeholders,” Antimicrobial Resistance & Infection Control 5 (2016): 20, 10.1186/s13756-016-0119-2. PMC4875594 27213040

[22] C. Vargas , J. Whelan , J. Brimblecombe , and S. Allender , “Co‐Creation, Co‐Design, Co‐Production for Public Health – a Perspective on Definition and Distinctions,” Public Health Research & Practice 32, no. 2 (2022): e3222211, 10.17061/phrp3222211. 35702744

[23] E. Blomkamp , “The Promise of Co‐Design for Public Policy,” Australian Journal of Public Administration 77, no. 4 (2018): 729–743, 10.1111/1467-8500.12310.

[24] J. Stier and S. E. Smit , “Co‐Creation as An Innovative Setting to Improve the Uptake of Scientific Knowledge: Overcoming Obstacles, Understanding Considerations and Applying Enablers to Improve Scientific Impact in Society,” Journal of Innovation and Entrepreneurship 10 (2021): 35, 10.1186/s13731-021-00176-2. PMC8475873 34603927

[25] N. Laurisz , M. Ćwiklicki , M. Żabiński , R. Canestrino , and P. Magliocca , “Co‐Creation in Health 4.0 as a New Solution for a New Era,” Healthcare 11, no. 3 (2023): 363, 10.3390/healthcare11030363. PMC9913923 36766938

[26] G. R. Longworth , K. Goh , D. M. Agnello , et al., “A Review of Implementation and Evaluation Frameworks for Public Health Interventions to Inform Co‐Creation: A Health Cascade Study,” Health Research Policy and Systems 22, (March 2024): 39, 10.1186/s12961-024-01126-6. PMC10976753 38549162

[27] Z. T. M. Scheefhals , E. F. de Vries , J. N. Struijs , M. E. Numans , and J. van Exel , “Stakeholder Perspectives on Payment Reform in Maternity Care in the Netherlands: A Q‐Methodology Study,” Social Science & Medicine ( 340 (January 2024): 116413, 10.1016/j.socscimed.2023.116413. 38000174

[28] Bundesamt für Gesundheit (BAG) . Forum Medizinische Grundversorgung [Internet], https://www.bag.admin.ch/de/forum-medizinische-grundversorgung.

[29] Universität Zürich . Citizens’ Assembly 2025 [Internet]. 2023, https://www.pnyx25.uzh.ch/en.html.

[30] C. Dingelstad , N. Hodyl , R. Wyse , S. Lewis , A. Koschel , and N. Goodwin , “Co‐Production, Co‐Creation and Co‐Design in Primary Health Care: A Scoping Review of Design, Implementation, Impact and Sustainability,” Health Expectations 28, no. 5 (2025): e70447, 10.1111/hex.70447. PMC12818046 41045116

[31] M. Bird , M. McGillion , E. M. Chambers , et al., “A Generative Co‐Design Framework for Healthcare Innovation: Development and Application of An End‐User Engagement Framework,” Research Involvement and Engagement 7(2021): 12, 10.1186/s40900-021-00252-7. PMC7923456 33648588

[32] S. F. M. Chastin , N. Smith , D. M. Agnello , et al., “Principles and Attributes of Evidence‐Based Co‐Creation: From Naïve Praxis Toward a Trustworthy Methodology ‐ A Health Cascade Study,” Public Health 248 (November 2025): 105922, 10.1016/j.puhe.2025.105922. 40992098

[33] P. Käppeli , N. Messerli , A. Romanova , S. Essig , and A. Balthasar «Health2040»: Innovative Angebote ambulanter Grundversorgung in der Schweiz − Bestandesaufnahme und Perspektive. Evidence Brief [Internet]. Luzern; 2024, https://www.health2040.ch/vision-action-plan/innovative-practices.

[34] C. Kaufmann , Z. Föhn , and A. Balthasar Zukünftige ambulante Grundversorgung: Einstellungen und Präferenzen der Bevölkerung (Obsan Bericht 04/2021) [Internet]. Neuchâtel; 2021 Sep., https://www.obsan.admin.ch/de/publikationen/2021-zukuenftige-ambulante-grundversorgung-einstellungen-und-praeferenzen-der.

[35] N. Amador‐Fernández , S. P. Jenkinson , and J. Berger , “Vision and Practice of Self‐Care for Community Pharmacy in Switzerland,” Exploratory Research in Clinical and Social Pharmacy 9 (March 2023): 100253, 10.1016/j.rcsop.2023.100253. PMC10121475 37095890

[36] T. Cartier , N. Senn , J. Cornuz , and Y. Bourgueil Switzerland. In: Building Primary Care in a Changing Europe: Case Studies [Internet] [Internet]. European Observatory on Health Systems and Policies; 2015 [cited 2023 Nov 6], https://www.ncbi.nlm.nih.gov/books/NBK459012/.

[37] R. Josi , M. Bianchi , and S. K. Brandt , “Advanced Practice Nurses in Primary Care in Switzerland: An Analysis of Interprofessional Collaboration,” BMC Nursing 19, no. 1 (January 2020): 1, 10.1186/s12912-019-0393-4. PMC6941298 31908597

[38] G. Nellessen‐Martens and W. Hoffmann Versorgungsforschung – eine Disziplin im Aufschwung. GGW ‐ Gesundheit + Gesellschaft Wissenschaft [Internet]. 2017, https://www.wido.de/fileadmin/Dateien/Dokumente/Publikationen_Produkte/GGW/wido_ggw_0117_nellessen_hoffmann.pdf.

[39] R. Kuhn , W. Konrad , S. K. Wist , and B. Witzel , Co‐Creation Toolkit: A Guidance on the Design, Development and Implementation of Effective Co‐creation in Industry‐Citizen Collaboration Settings [Internet] (DIALOGIK gemeinnützige Gesellschaft für Kommunikations‐ und Kooperationsforschung mbH, 2021), https://www.ssoar.info/ssoar/handle/document/72916.

[40] S. Lee and M. E. Sosa , “Spaces for Creativity: Unconventional Workspaces and Divergent Thinking,” Management Science 72, no. 2 (2025): 1072–1094, 10.1287/mnsc.2022.02052.

[41] K. Thoring , M. Guerreiro Goncalves , R. M. Mueller , P. Badke‐Schaub , and P. Desmet , Inspiration Space: Towards a Theory of Creativity‐Supporting Learning Environments, eds. E. Bohemia , C. de Bont , and L. Svengren Holm (The Design Society, 2017. 5, 1539–1561. (Conference Proceedings of the Design Management Academy), http://resolver.tudelft.nl/uuid:598d060f-591c-444e-833b-999c47651464.

[42] A. Tanner Swiss Academies of Arts and Sciences: Td‐Net Toolbox for Co‐Producing Knowledge. [Internet]. 2021. Tell your Story by Means of an Object, https://zenodo.org/records/4627120#.YHftN2hCTBI.

[43] A. Berditchevskaia and C. Bertoncin How to Make Good Group Decisions [Internet]. Nesta; 2021, https://media.nesta.org.uk/documents/Collective-Intelligence-Good-Decision-Making.pdf.

[44] H. Brouwer and J. Brouwers The MSP Tool Guide: Sixty Tools to Facilitate Multi‐Stakeholder Partnerships [Internet]. Centre for Development Innovation, Wageningen University and Research; 2017, https://mspguide.org/the-msp-tool-guide/.

[45] Living Guide to Social Innovation Labs . Systems Mapping [Internet], https://mars-solutions-lab.gitbook.io/living-guide-to-social-innovation-labs/seeing/understanding-the-problem-systems-and-complexity/systems-mapping.

[46] P. Bate and G. Robert , Bringing User Experience to Healthcare Improvement: The Concepts, Methods and Practices of Experience‐Based Design (Radcliffe Publishing, 2007).

[47] G. Silvestri , G. Diercks , and C. Matti . X‐CURVE booklet. A sensemaking tool to foster collective narratives on system change [Internet]. 2022, https://transitionshub.climate-kic.org/publications/x-curve-a-sensmaking-tool-to-foster-collective-narratives-on-system-change/.

[48] K. A. Laugaland , K. Akerjordet , C. T. Frøiland , and I. Aase , “Co‐Creating Digital Educational Resources to Enhance Quality in Student Nurses’ Clinical Education in Nursing Homes: Report of a Co‐Creative Process,” Journal of Advanced Nursing 79, no. 10 (2023): 3899–3912, 10.1111/jan.15800. 37461247

[49] E. Marín‐González , D. Malmusi , L. Camprubí , and C. Borrell , “The Role of Dissemination as a Fundamental Part of a Research Project: Lessons Learned From SOPHIE,” International Journal of Health Services 47, no. 2 (April 2017): 258–276, 10.1177/0020731416676227. 27799595

[50] Gesundheitsversorgung der Zukunft – Was bringt Health 2040, Manuela Oetterli? [Internet]. 2025, https://www.luzerner-forum.ch/gesundheitsversorgung-der-zukunft---was-bringt-health-2040,-manuela-oetterli.

[51] A. Balthasar , M. Oetterli , A. Romanova , P. Käppeli , and S. Mantwill «Gesundheitsnetz für alle – pour tous – per tutti»: Aktionsplan für eine zukunftsfähige ambulante Grundversorgung in der Schweiz. Forschungsprojekt «Health2040» der Universität Luzern und Interface Politikstudien Forschung Beratung AG [Internet]. 2024, https://www.health2040.ch/vision-action-plan/results-reports.

[52] Bundesamt für Gesundheit (BAG) , Agenda Grundversorgung. Fachbericht der Steuergruppe zuhanden des Eidgenössischen Departements des Innern [Internet] (Bundesamts für Gesundheit (BAG), 2025), https://www.bag.admin.ch/de/agenda-grundversorgung#Weitere-Informationen.

[53] Stadt Luzern . Stadt Luzern startet Pilotprojekt für integrierte Gesundheitsversorgung [Internet]. 2026, https://www.stadtluzern.ch/aktuelles/newslist/2980447.

[54] Stadt Luzern . Gesundheitswegweiser Luzern [Internet]. 2026, https://www.stadtluzern.ch/projekte/soziales/99235.

[55] B. W. Head , Wicked Problems in Public Policy: Understanding and Responding to Complex Challenges [Internet] (Springer International Publishing, 2022), 10.1007/978-3-030-94580-0.

[56] WHO ., Integrated Care Models: An Overview: Working Document [Internet] (World Health Organization. Regional Office for Europe, 2016), https://iris.who.int/items/ba7389a2-6ac5-4cb1-803d-75e1ac6d8f00.

[57] L. N. Allen , K. Rasanathan , R. Mash , M. V. Uribe , V. Martinez‐Bianchi , and M. Kidd , “Models of Global Primary Care Post‐2030,” Lancet Primary Care 1, no. 3 (September 2025): 100027, 10.1016/j.lanprc.2025.100027. PMC12485077 41040968

[58] B. Starfield , Primary Care: Balancing Health Needs, Services, and Technology [Internet] (Oxford University Press, 1998), 10.1093/oso/9780195125429.001.0001.

[59] D. M. Agnello , V. Anand‐Kumar , Q. An , et al., “Co‐Creation Methods for Public Health Research — Characteristics, Benefits, and Challenges: A Health Cascade Scoping Review,” BMC Medical Research Methodology 25, no. 1 (March 2025): 60, 10.1186/s12874-025-02514-4. PMC11884017 40050729

[60] T. Ø. Bentzen , “Continuous Co‐Creation: How Ongoing Involvement Impacts Outcomes of Co‐Creation,” Public Management Review 24, no. 1 (January 2022): 34–54, 10.1080/14719037.2020.1786150.

[61] T. Pearce , M. Maple , A. Shakeshaft , S. Wayland , and K. McKay , “What Is the Co‐Creation of New Knowledge? A Content Analysis and Proposed Definition for Health Interventions,” International Journal of Environmental Research and Public Health 17, no. 7 (January 2020): 2229, 10.3390/ijerph17072229. PMC7177645 32224998

[62] M. T. E. Puts , S. Sattar , V. Ghodraty‐Jabloo , et al., “Patient Engagement in Research With Older Adults With Cancer,” Journal of Geriatric Oncology 8, no. 6 (November 2017; Geriatric Oncology and Supportive Care: Highlights from the 2016 International Society of Geriatric Oncology Annual Conference): 391–396, 10.1016/j.jgo.2017.05.002. 28599854

[63] P. Slattery , A. K. Saeri , and P. Bragge , “Research Co‐Design in Health: A Rapid Overview of Reviews,” Health Research Policy and Systems 18, no. 1 (February 2020): 17, 10.1186/s12961-020-0528-9. PMC7014755 32046728

[64] T. L. McCarron , T. Noseworthy , K. Moffat , et al., “A Co‐Designed Framework to Support and Sustain Patient and Family Engagement in Health‐Care Decision Making,” Health Expectations 23, no. 4 (2020): 825–836, 10.1111/hex.13054. PMC7495064 32337836

[65] D. E. Miranda , R. Mead , B. Soto‐Ponce , M. Mikulak , L. Orton , and S. Scott , “The Role of Power in Co‐Approaches to Health Research: Insights From Spain and the United Kingdom With a Rapid Review of Reviews,” Health Expectations 28, no. 5 (2025): e70381, 10.1111/hex.70381. PMC12539374 41118204

[66] M. Roura , “The Social Ecology of Power in Participatory Health Research,” Qualitative Health Research 31, no. 4 (March 2021): 778–788, 10.1177/1049732320979187. 33349170

[67] J. de Boer , G. R. Longworth , L. R. Delfmann , et al., “Exploring Co‐Adaptation for Public Health Interventions: Insights From a Rapid Review and Interviews,” BMC Public Health 25, no. 1 (February 2025): 614, 10.1186/s12889-025-21544-7. PMC11827302 39953479

