# Supplementary material for: Co‐Creation for Health System Innovation: Developing a Model and an Action Plan for Future Primary Care in Switzerland
Source: Health Expect. 2026 Oct 22;29(5):e70927. doi: 10.1111/hex.70927 (PMC13688086; doi:10.1111/hex.70927)
Supplement: Supplementary file 1 [file HEX-29-e70927-s001.pdf]

Model Building Blocks

An important foundation for the co-creation primary care models for the Swiss population were the so-called “model building blocks.” Based on the Health2040 sub-studies and the analysis of existing innovative care models, eleven building blocks and their corresponding variants were defined. Additional blocks and choice options could be added during Workshop I if needed. An additional building block (“Joker”) was available for further creative and innovative ideas.

Overview of the model building blocks for the co-creation of primary care models

	Building Block	*Description*	*Options*
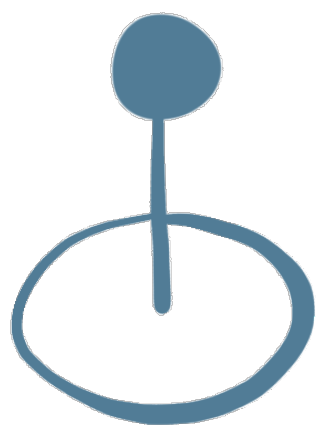	1. Location	Location of the care model	– Entire Switzerland<br>– German-speaking Switzerland<br>– French-speaking Switzerland<br>– Ticino
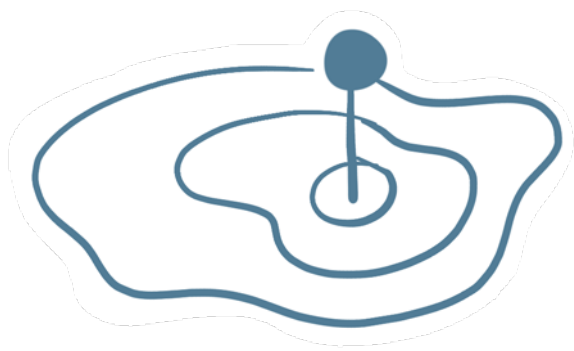	2. Scope	Area in which the care model provides services	– Local<br>– Regional<br>– National

	Building Block	*Description*	*Options*
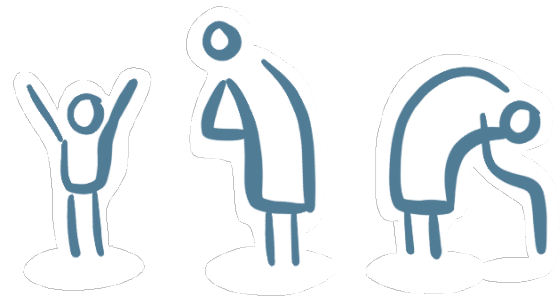	3. Target Group	Target group addressed by the care model's services	– General population<br>– Older adults<br>– People with chronic conditions
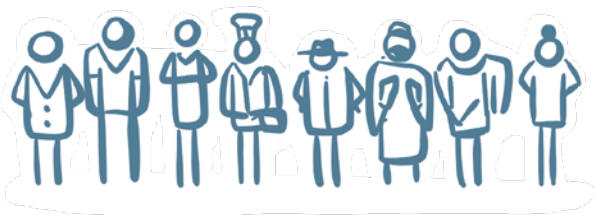	4. Involved Service Providers	Service providers involved in the care model and serving the target group	– General practitioners (GPs)<br>– Physician specialists<br>– Nurses<br>– Advanced Practice Nurses (APN)<br>– Health and long-term care professionals<br>– Pharmacists<br>– Medical practice assistants (MPA) / Medical practice coordinators (MPK)<br>– Social workers<br>– Therapeutic professions<br>– Counseling professions<br>– Support services<br>– Gastronomy<br>– Group "Neighbors, volunteers, and relatives"

	Building Block	*Description*	*Options*
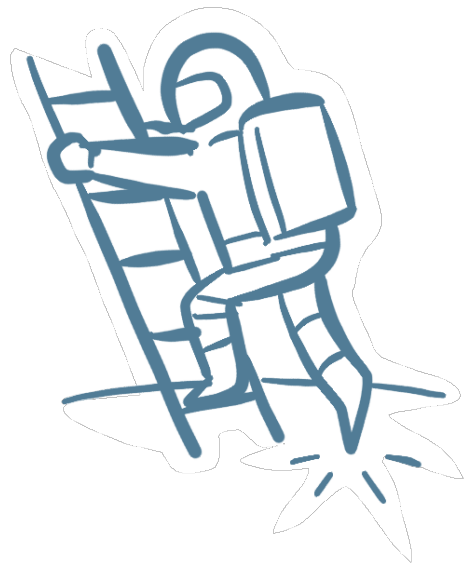	5. First Contact	*First Contact Infrastructure*<br>Spatial setting in which first contact takes place	– GP practice<br>– Hospital emergency department<br>– Pharmacy<br>– Health center<br>– Community center<br>– Online/telephone<br>– At the person's home
		First Contact Responsibility<br>Professional group responsible for first contact with the target group	– General practitioners<br>– Nurses<br>– Advanced Practice Nurses (APN)<br>– Health and long-term care professionals<br>– Pharmacists<br>– Medical practice assistants (MPA) / Medical practice coordinators (MPK)<br>– Social workers<br>– Therapeutic professions<br>– Counseling professions<br>– Via an app
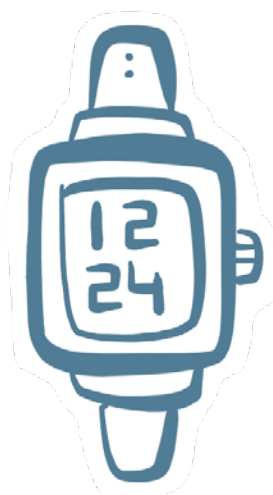	6. Opening Hours	Opening hours of the care model	– Office hours (9:00–18:00)<br>– Extended office hours (7:00–22:00)<br>– 24/7 operation

Building Block	*Description*	*Options*
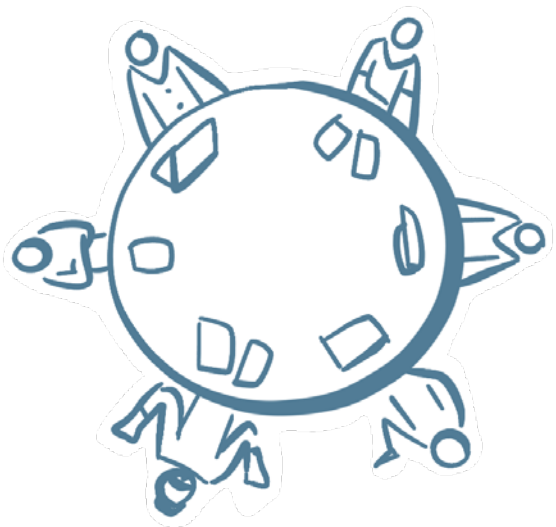 7. Coordination of Service Delivery	*Coordination Infrastructure*<br>Spatial setting in which coordination of services takes place	– GP practice<br>– Pharmacy<br>– Health center<br>– Community center<br>– Independent coordination office<br>– Inpatient institution
	Coordination Responsibility<br>Professional group responsible for coordination of services	– General practitioners<br>– Nurses<br>– Advanced Practice Nurses (APN)<br>– Health and long-term care professionals<br>– Pharmacists<br>– Medical practice assistants (MPA) / Medical practice coordinators (MPK)<br>– Social workers<br>– Therapeutic professions<br>– Counseling professions

	Building Block	*Description*	*Options*
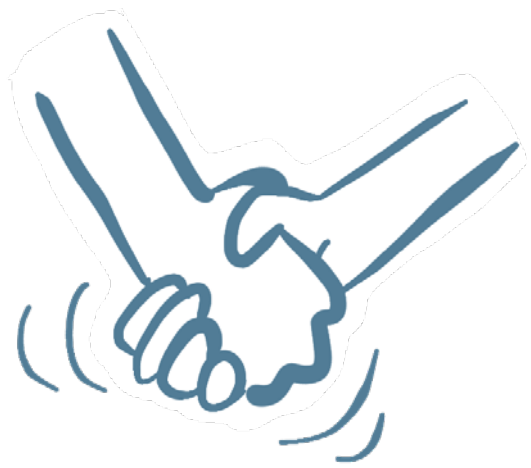	8. Treatment and Support	*Treatment & Support Infrastructure*<br>Spatial setting in which treatment and support of patients takes place	– GP practice<br>– Pharmacy<br>– Health center<br>– Community center<br>– Inpatient institution<br>– At the person's home
		Responsibility for Treatment Plan<br>Professional group responsible for the treatment plan	– General practitioners<br>– Physician specialists<br>– Nurses<br>– Advanced Practice Nurses (APN)<br>– Health and long-term care professionals<br>– Pharmacists
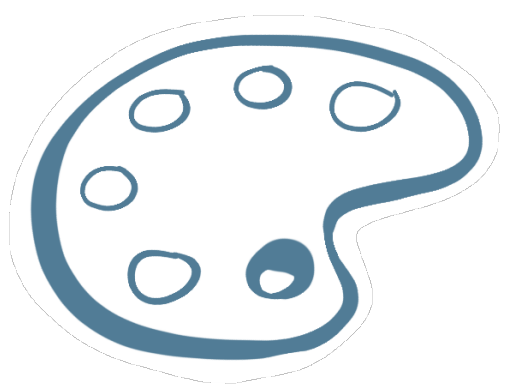	9. Services Provided	Scope of services provided by the care model	– Acute care<br>– Treatment of chronic conditions<br>– Prevention<br>– Inpatient services<br>– Social counseling<br>– Everyday life support
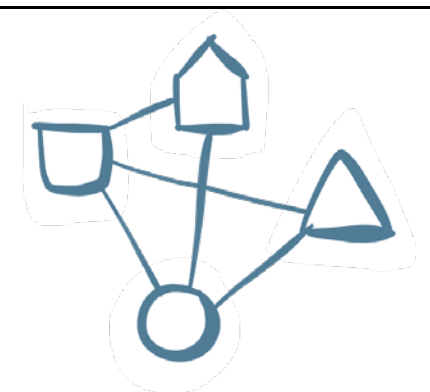	10. Organizational Form	Organizational form of the care model	– Extended GP practice<br>– Multiprofessional health center<br>– Health cooperative<br>– Health community

	Building Block	*Description*	*Options*
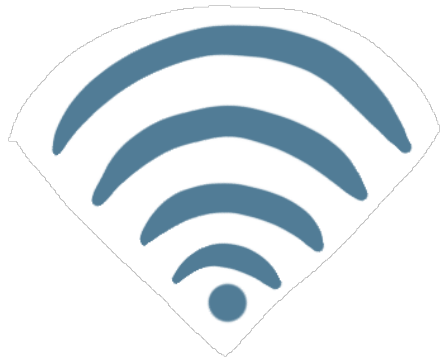	11. Integration of Digital Technologies	Use of digital technologies in the care model	To be considered when defining the other building blocks
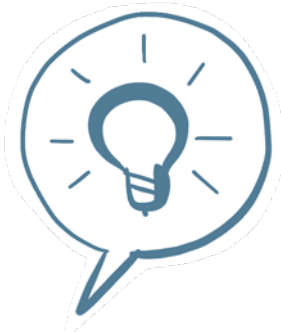	12. Joker	Additional creative ideas	No fixed variants (e.g., type of service delivery)

Modellbausteine

Joker
Zusätzliche kreative Ideen

Standort
Standort des Versorgungsmodells

- Ganze Schweiz
- Westschweiz
- Deutschschweiz
- Tessin

Radius
Raum, für den das Versorgungsmodell Leistungen erbringt.

- Lokal
- Regional
- National

Zielgruppe
Zielgruppe, an die sich die Leistungen des Versorgungsmodells richten.

- Allgemeine Bevölkerung
- Ältere Personen
- Personen mit chronischen Erkrankungen

Beteiligte Leistungserbringende
Leistungserbringende, die am Versorgungsangebot beteiligt sind und die Zielgruppe versorgen.

- Hausärzte/-innen
- Fachärzte/-innen
- Pflegefachpersonen
- Pflegeexperten/-innen APN
- Fachpersonen Gesundheit/Betreuung
- Apotheker/-innen
- Medizinische Praxisassistenten/-innen MPA / Medizinische Praxiskoordinatoren/-innen MPK
- Sozialarbeiter/-innen
- Therapeutische Professionen
- Beratende Professionen
- Unterstützungsdienste
- Gastronomie
- Gruppe «Nachbarn/-innen, Freiwillige, Angehörige und Patientenvertretungen»

Öffnungszeiten
Öffnungszeiten des Versorgungsmodells

- Bürozeiten (9.00–18.00 Uhr)
- Verlängerte Bürozeiten (7.00–22.00 Uhr)
- Betrieb rund um die Uhr

Erstkontakt

Infrastruktur
Räumlicher Kontext, in dem der Erstkontakt stattfindet.

- Hausarztpraxis
- Apotheke
- Notfallstation eines Spitals
- Gesundheitszentrum
- Online/Telefon
- Gemeinschaftszentrum
- Bei der Person zu Hause

Verantwortung
Berufsgruppe, die für die erste Kontaktaufnahme mit der Zielgruppe verantwortlich ist.

- Hausärzte/-innen
- Pflegefachpersonen
- Pflegeexperten/-innen APN
- Fachpersonen Gesundheit/Betreuung
- Apotheker/-innen
- Über eine App
- Medizinische Praxisassistenten/-innen MPA / Medizinische Praxiskoordinatoren/-innen MPK
- Therapeutische Professionen
- Beratende Professionen
- Sozialarbeiter/-innen

Integration Digitaler Technologien
Einsatz digitaler Technologien im Versorgungsmodell

- Berücksichtigung bei Bestimmung der übrigen Bausteine

Koordination der Leistungserbringung

Infrastruktur
Räumlicher Kontext, in dem die Koordination der Leistungserbringung stattfindet.

- Hausarztpraxis
- Gesundheitszentrum
- Apotheke
- Gemeinschaftszentrum
- Eigenständige Koordinationsstelle
- Stationäre Institution

Verantwortung
Berufsgruppe, die für die Koordination der Leistungserbringung verantwortlich ist.

- Hausärzte/-innen
- Pflegefachpersonen
- Pflegeexperten/-innen APN
- Fachpersonen Gesundheit/Betreuung
- Apotheker/-innen
- Sozialarbeiter/-innen
- Medizinische Praxisassistenten/-innen MPA / Medizinische Praxiskoordinatoren/-innen MPK
- Therapeutische Professionen
- Beratende Professionen

Organisationsform
Zugrunde liegende Organisationsform des Versorgungsmodells

- Erweiterte Hausarztpraxis
- Multiprofessionelles Gesundheitszentrum
- Gesundheitskooperation
- Gesundheitsgemeinschaft

Behandlung und Unterstützung

Infrastruktur
Räumlicher Kontext, in dem die Behandlung und Unterstützung der Betroffenen stattfindet.

- Hausarztpraxis
- Gesundheitszentrum
- Gemeinschaftszentrum
- Apotheke
- Stationäre Institution
- Bei der Person zu Hause

Verantwortung
Berufsgruppe, die für den Behandlungsplan verantwortlich ist.

- Hausärzte/-innen
- Fachärzte/-innen
- Pflegefachpersonen
- Apotheker/-innen
- Pflegeexperten/-innen APN
- Fachpersonen Gesundheit/Betreuung

Angebotsintern erbrachte Leistungen
Umfang der Leistungen, die das Versorgungsmodell erbringt.

- Akutbehandlung
- Prävention
- Behandlung chronischer Erkrankungen
- Stationäre Angebote
- Entlastung im Alltag
- Sozialberatung

Patient Profiles

Another important foundation of the co-creation process was patient profiles. These represented key population groups in outpatient primary care. Based on the five patient profiles below, participants developed models of primary care in Workshop I. In addition, patients participated in the workshop and enriched the profiles with their personal experiences. Profile descriptions were also supplemented with insights from the literature on preferences of these groups towards future primary care.

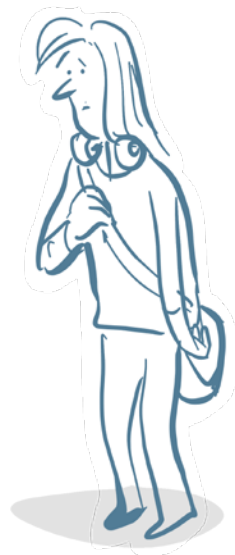

Person 1: Person 1: A young adult living in the city, seeking medical advice for non-specific symptoms such as fatigue. Signs of depression and dizziness are present.

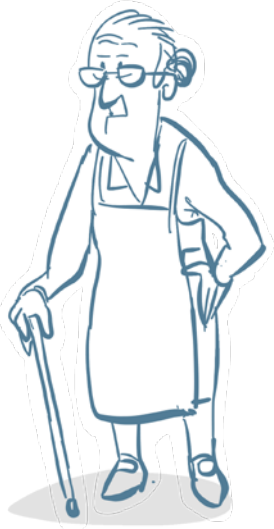

Person 2: An older person living in a rural area. They suffer from several chronic conditions and occasionally require home care and treatment by a specialist.

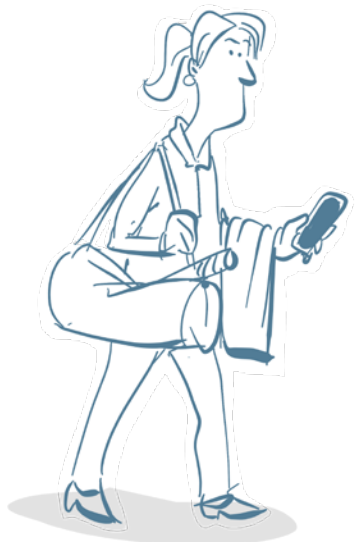

Person 3: A middle-aged working person (40 years old) with an active lifestyle seeks medical help in an acute health situation. They have had fever and severe upper abdominal pain for several days.

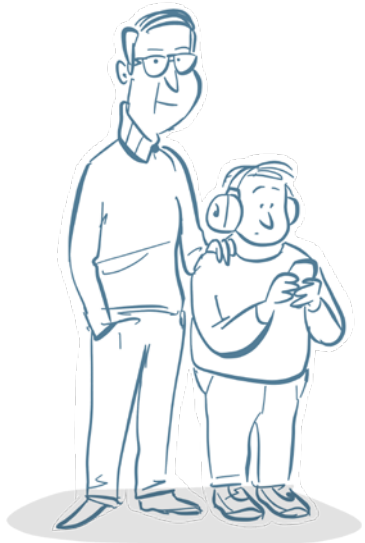

Person 4: A parent with a 12-year-old child seeks care in a non-acute situation. The child shows behavioral problems and is overweight. The parent is concerned about the financial consequences of potential treatment.

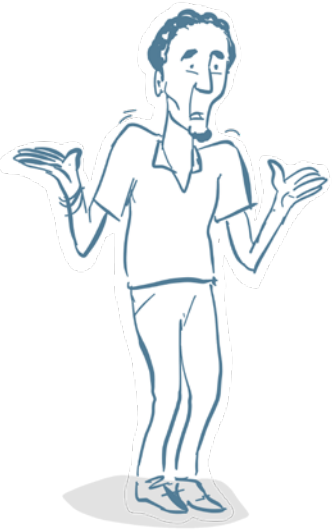

Person 5: A person with a migration background has difficulties with the local Swiss languages. They present with a slightly elevated body temperature of 38°C but no other symptoms. Nevertheless, they are worried about their condition.

Person 1

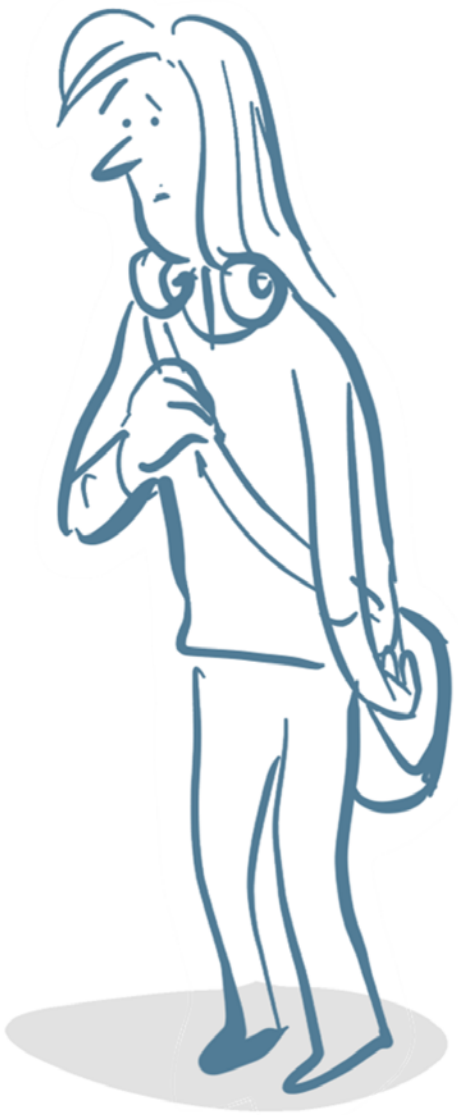

Eine junge, erwachsene Person, die in der Stadt wohnt und medizinischen Rat bei unspezifischen Symptomen wie Müdigkeit sucht. Es zeigen sich Anzeichen von Depressionen und Schwindelgefühle.

Hintergrundwissen:

Welche Präferenzen haben junge Personen in der medizinischen Versorgung gemäss aktuellen Studien?

- Sie bevorzugen den Hausarzt oder die Hausärztin für medizinische Behandlungen, sind aber offen gegenüber alternativen Optionen wie z.B. mobile Gesundheits-Apps, qualifiziertes Fachpersonal und Apotheken.
- Sie legen Wert auf kurze Wartezeiten. Eine schnelle Terminvergabe ist für sie weniger wichtig.
- Sie schätzen erweiterte Praxisöffnungszeiten.
- Sie erachten es als wichtig, dass Fachpersonen Zugang zu den Patientendossiers haben. Persönlicher Kontakt in der laufenden Betreuung ist weniger wichtig.
- Sie sind preissensibler.

Person 2

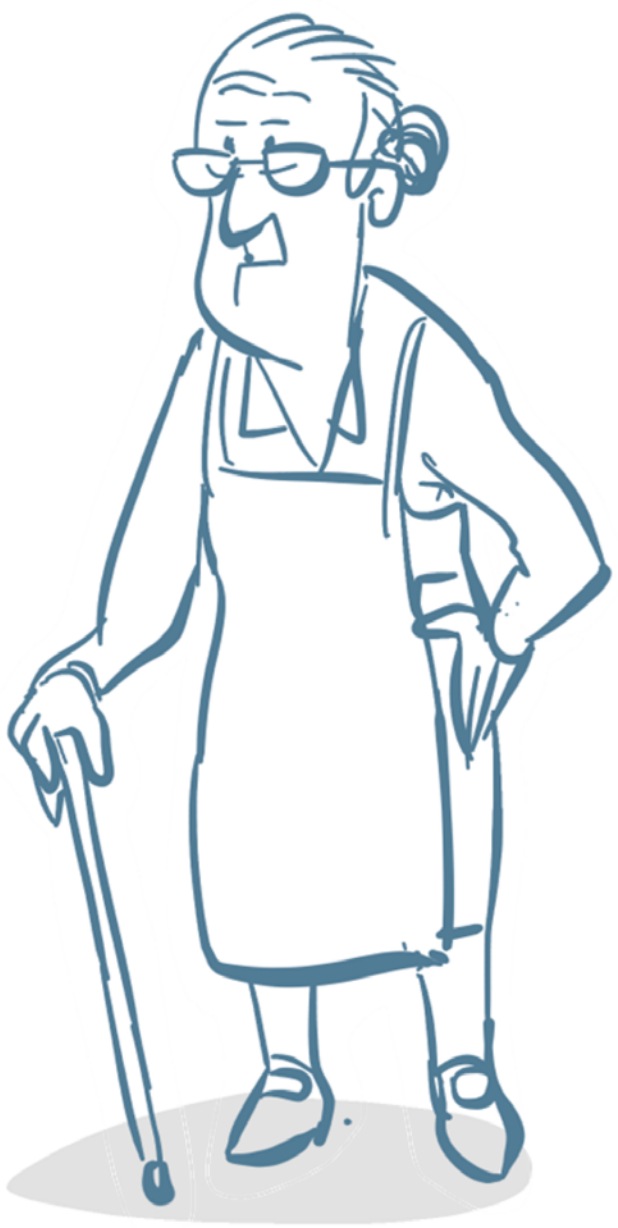

Eine ältere Person, die in einer ländlichen Gegend lebt. Sie leidet unter mehreren chronischen Krankheiten und benötigt manchmal häusliche Pflege und Behandlung von einer Spezialistin/einem Spezialisten.

Hintergrundwissen:

Welche Präferenzen haben ältere Personen mit chronischen Krankheiten in der medizinischen Versorgung gemäss aktuellen Studien?

- Sie bevorzugen den Hausarzt oder die Hausärztin als primäre Ansprechperson und als Koordinator/-in der Gesundheitsversorgung. Pflege durch Pflegeexperten/-innen APN wird angenommen, insbesondere bei akuten Gesundheitsproblemen.
- Sie schätzen persönliche Interaktionen und halten es für wichtig, dass Gesundheitsdienstleistende Zugang zur medizinischen Vorgeschichte haben.
- Sie bevorzugen traditionelle Praxisöffnungszeiten.
- Sie sind offen gegenüber alternativer Medizin.
- Sie sind skeptisch gegenüber digitalen Gesundheitsangeboten und haben eine niedrigere Gesundheitskompetenz.
- Sie haben Vertrauen in das Urteil von Fachpersonen. Gemeinsame Entscheidungsfindung ist keine Notwendigkeit.
- Sie benötigen Unterstützungsangebote vor Ort, die den Aufbau sozialer Kontakte ermöglichen.

Person 3

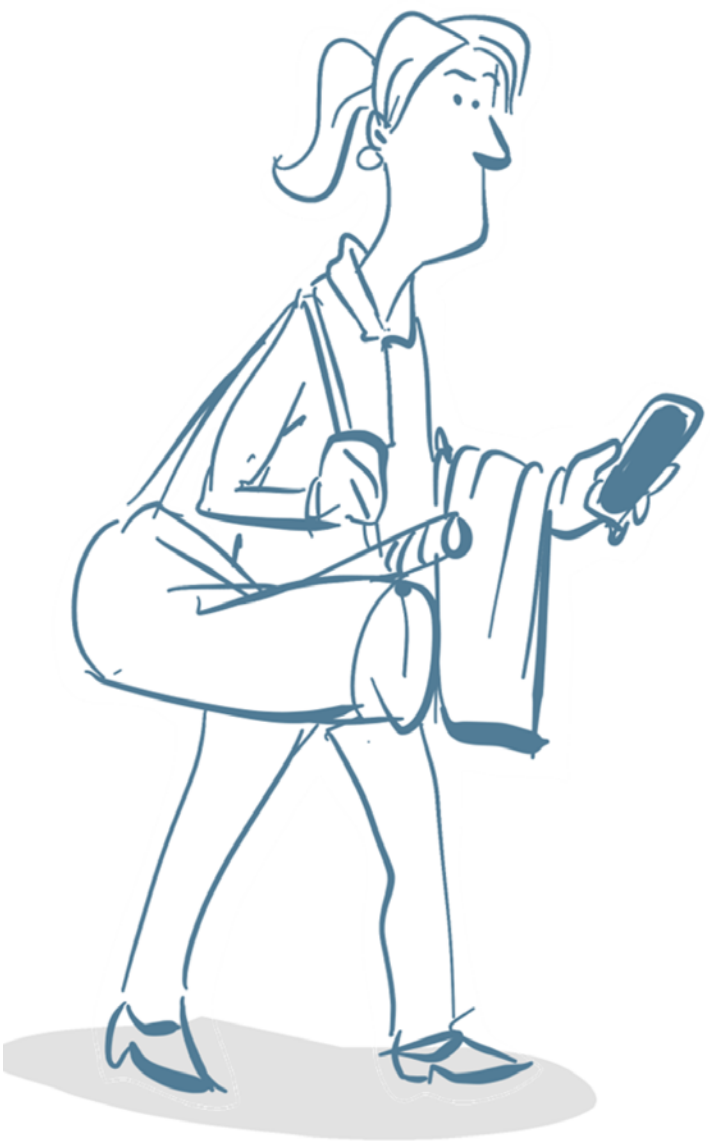

Eine berufstätige Person mittleren Alters (40 Jahre alt) mit einem aktiven Lebensstil sucht in einer akuten Gesundheitssituation ärztliche Hilfe. Sie leidet seit mehreren Tagen an Fieber und starken Oberbauchschmerzen.

Hintergrundwissen:

Welche Präferenzen haben Personen mittleren Alters in der medizinischen Versorgung gemäss aktuellen Studien?

- Sie haben Interesse an praktischen Telemedizin-Optionen.
- Sie haben ein positives Bild von Apothekern/-innen als erste Ansprechpersonen im Gesundheitswesen.
- Sie akzeptieren Behandlungen durch Pflegefachpersonen.
- Sie bevorzugen erweiterte Öffnungszeiten bei Hausarztpraxen.
- Sie sind aufgeschlossen gegenüber verschiedenen digitalen Gesundheitsangeboten, inklusive Apps.
- Sie erachten die gemeinsame Entscheidungsfindung als wichtig.

Person 4

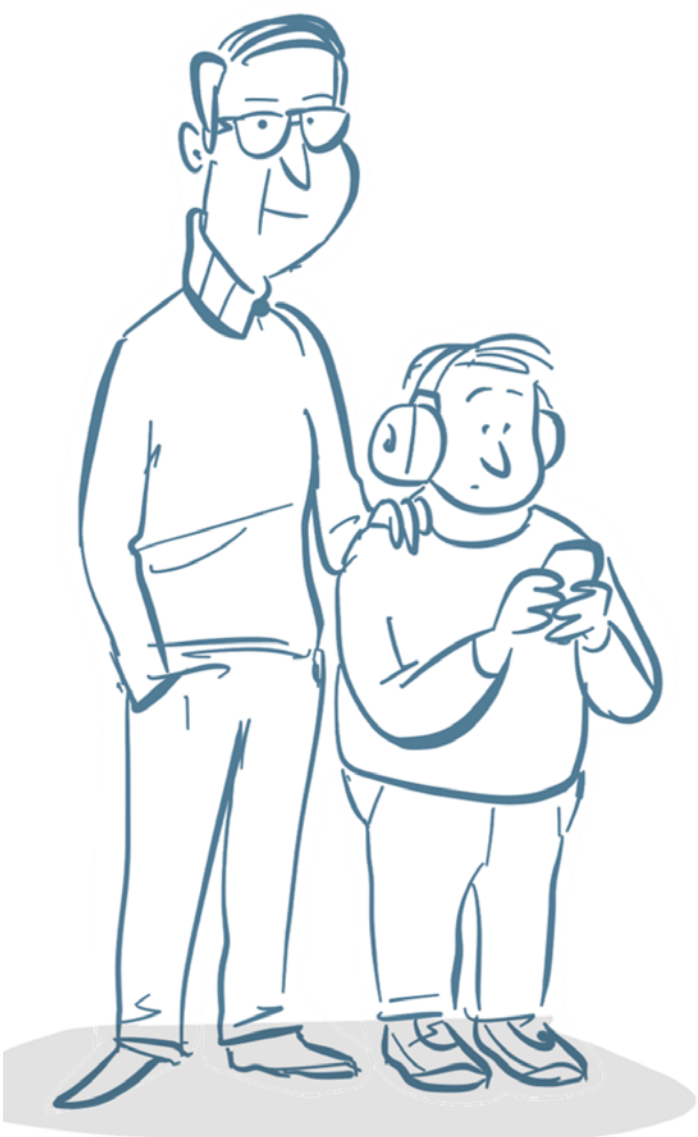

Ein Elternteil mit einem 12-jährigen Kind sucht Versorgung in einer nicht-akuten Situation. Das Kind zeigt Verhaltensprobleme und ist übergewichtig. Der Elternteil macht sich Sorgen über die finanziellen Konsequenzen einer allfälligen Behandlung.

Hintergrundwissen:

Welche Präferenzen haben Personen mit niedrigem Einkommen in der medizinischen Versorgung gemäss aktuellen Studien?

- Sie bevorzugen die Hausärztin oder den Hausarzt als Hauptbehandlungs- und Koordinationsinstanz und sind skeptisch gegenüber anderen Fachpersonen wie Pflegefachpersonen oder Apothekern/-innen.
- Sie erachten Hausarztpraxen mit Öffnungszeiten rund um die Uhr als wichtig.
- Sie sind skeptisch gegenüber digitalen Innovationen im Gesundheitswesen.
- Sie haben eine eher niedrige Gesundheitskompetenz.
- Sie vertrauen häufig den Entscheidungen von Fachpersonen bei wichtigen Gesundheitsfragen.

Person 5

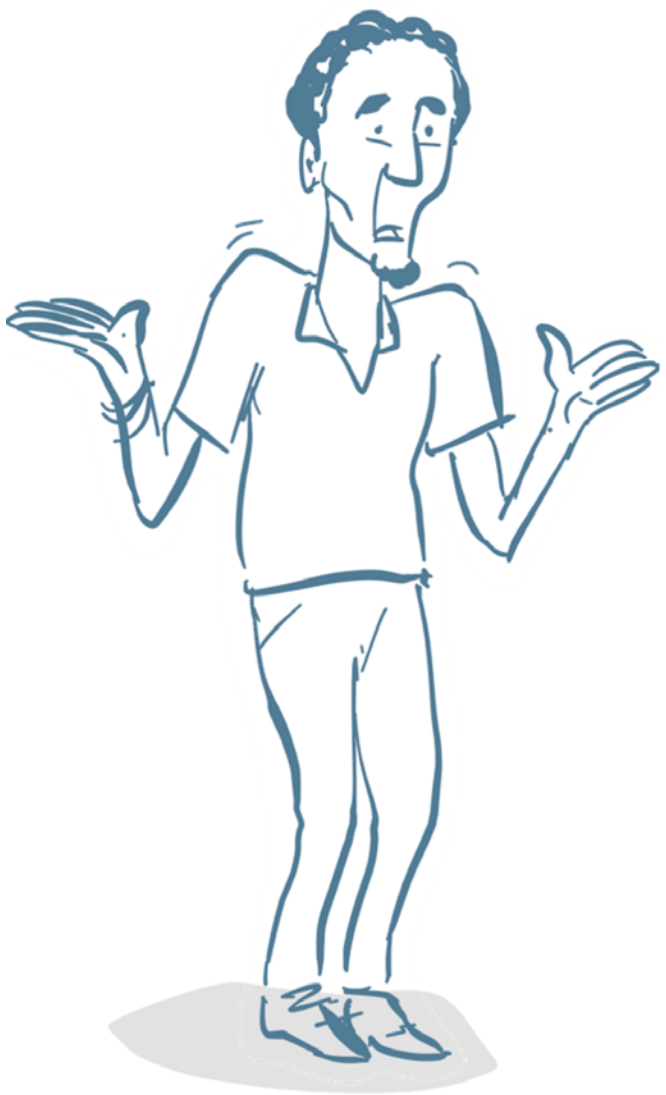

Eine Person mit Migrationshintergrund hat Schwierigkeiten mit den lokalen schweizerischen Sprachen. Sie weist eine leicht erhöhte Körpertemperatur von 38°C auf, zeigt jedoch keine weiteren Symptome. Trotzdem ist sie besorgt über ihren Zustand.

Hintergrundwissen:

Welche Präferenzen haben Personen mit Migrationshintergrund in der medizinischen Versorgung gemäss aktuellen Studien?

- Sie suchen eher die Notaufnahme als erste Anlaufstelle auf, auch ohne akute Symptome, als sich an eine Hausärztin oder einen Hausarzt zu wenden.
- Sie bevorzugen Gesundheitsdienstleistende, die auf Englisch kommunizieren können oder Übersetzungsdienste bereitstellen.
- Sie kennen sich nicht genügend im Schweizer Gesundheitssystem aus.
- Sie benötigen Unterstützungsangebote wie z.B. Gesundheitsinformationen in Englisch oder in ihrer Muttersprache, Gemeinschaftsunterstützung (durch Gemeinde, Quartier etc.) sowie kultursensible Betreuung.

X-Curve Canvas

Action Area X. ____________

Group _____

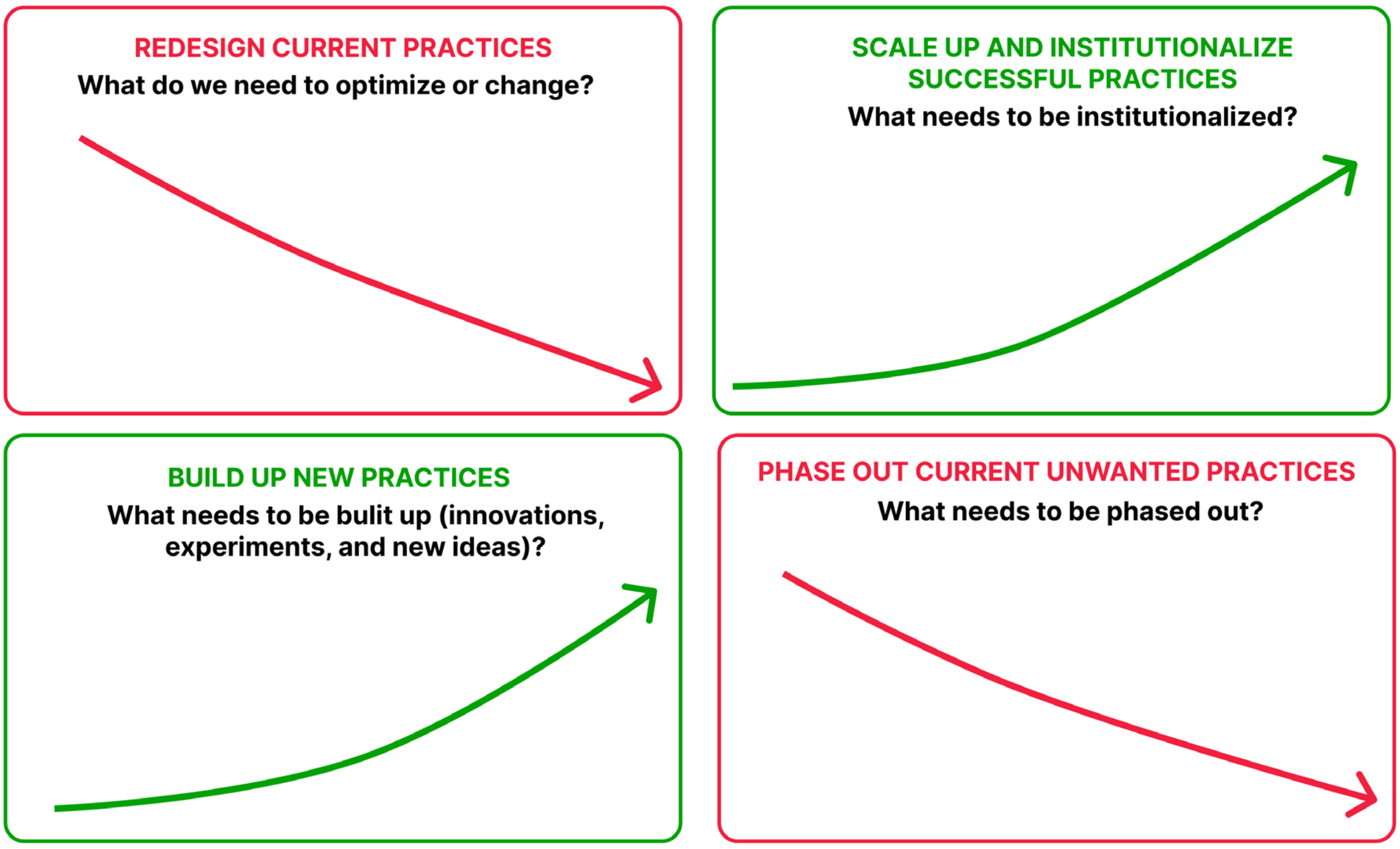

Action Plan Canvas

Professional group: __________

Action area I: ______________	Action area II: ______________	Action area III: ______________
Framework conditions:	Framework conditions:	Framework conditions:
Activities:	Activities:	Activities:
