# Supplementary material for: Co‐Creation for Health System Innovation: Developing a Model and an Action Plan for Future Primary Care in Switzerland
Source: Health Expect. 2026 Oct 22;29(5):e70927. doi: 10.1111/hex.70927 (PMC13688086; doi:10.1111/hex.70927)
Supplement: Supplementary file 2 [file HEX-29-e70927-s002.pdf]

Supplementary File 3. Examples of intermediate workshop outputs and their contribution to the final model and Action Plan

Workshop activity/source	Illustrative workshop artifact	Key ideas generated	Contribution
**Workshop I: patient-specific model development**	*Model developed for the young patient profile* 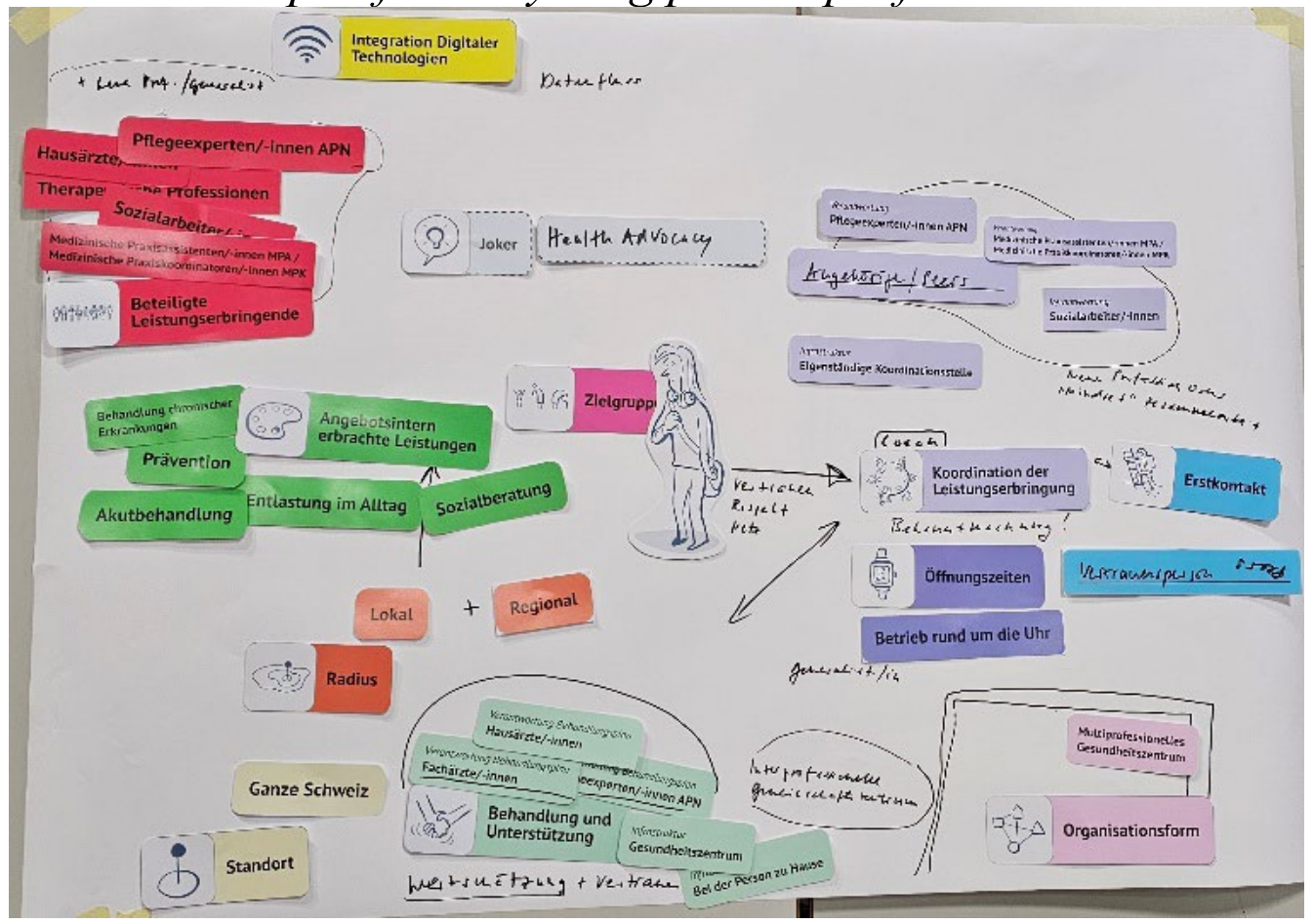	Multiprofessional health center bringing different services together; a new coordinating professional as first point of contact; regional organization of care with health centers connected to external services through a wider network; support through peers, nurses/medical assistants, home visits, and community resources; "health advocacy" added through the open joker.	Recurring and patient-specific elements informed the synthesized primary care model
	Model developed for the older patient profile 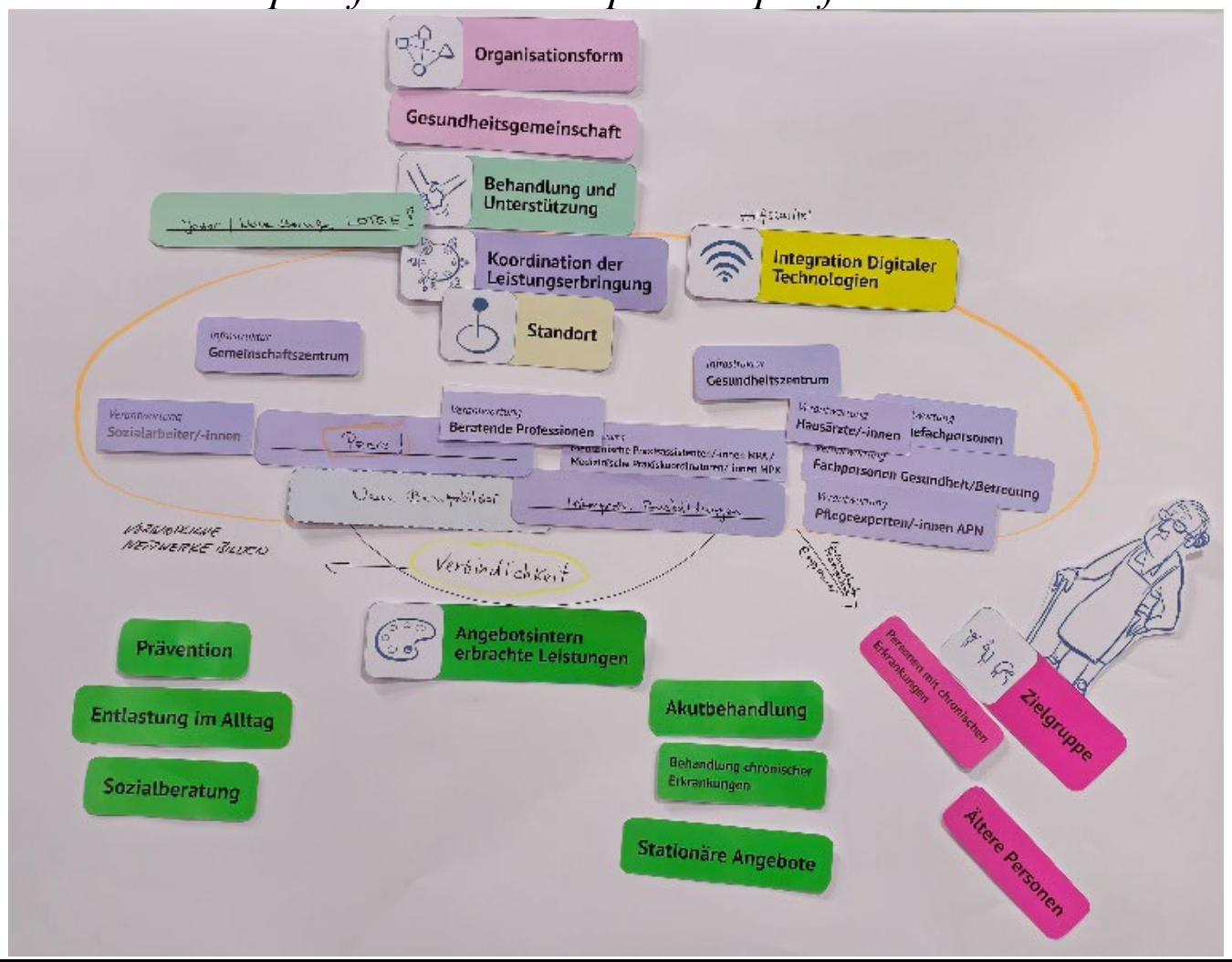	Multiprofessional health center as a geographically accessible one-stop shop for health, particularly for older people in rural areas with chronic conditions and broader social needs; broad understanding of health extending beyond medical treatment to social support, everyday assistance, and prevention; a new coordinator role proposed as a central element to help patients navigate the system; stronger networks and commitment between stakeholders, so that responsibility for follow-up would not rest solely with the patient; peer support to address experiential and social needs.	

<table>
<tr>
<td rowspan="2">Workshop I: model synthesis session</td>
<td>Synthesized model 3, "Fit durchs Leben" (Fit through Life)
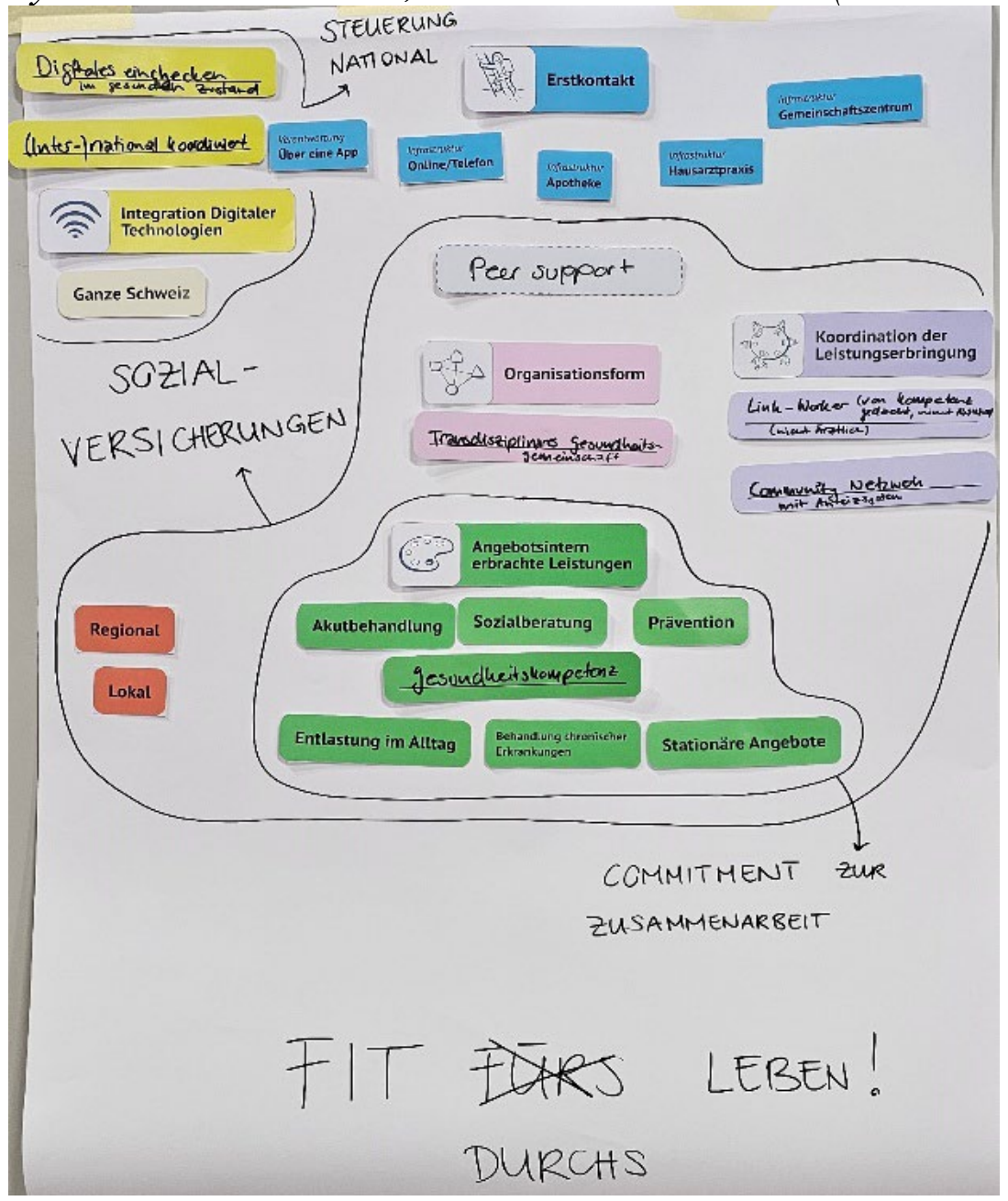
</td>
<td>Nationwide network of digitally connected health centers organized locally or regionally; multiple access points (app, telephone, pharmacy, general practice, community center); a coordination team combining health and social care functions; new navigation roles such as a link worker; comprehensive medical and social support, with attention to health literacy and prevention.

Proposed Action Areas: national coordination; consideration of a unified insurance model; and commitment to collaboration across silos, political levels, and cantonal boundaries.</td>
<td rowspan="2">Informed the overarching model and provided the Action Areas taken forward to Workshop II</td>
</tr>
<tr>
<td>Synthesized model 5, "Gesundheit für alle" (Health for All)
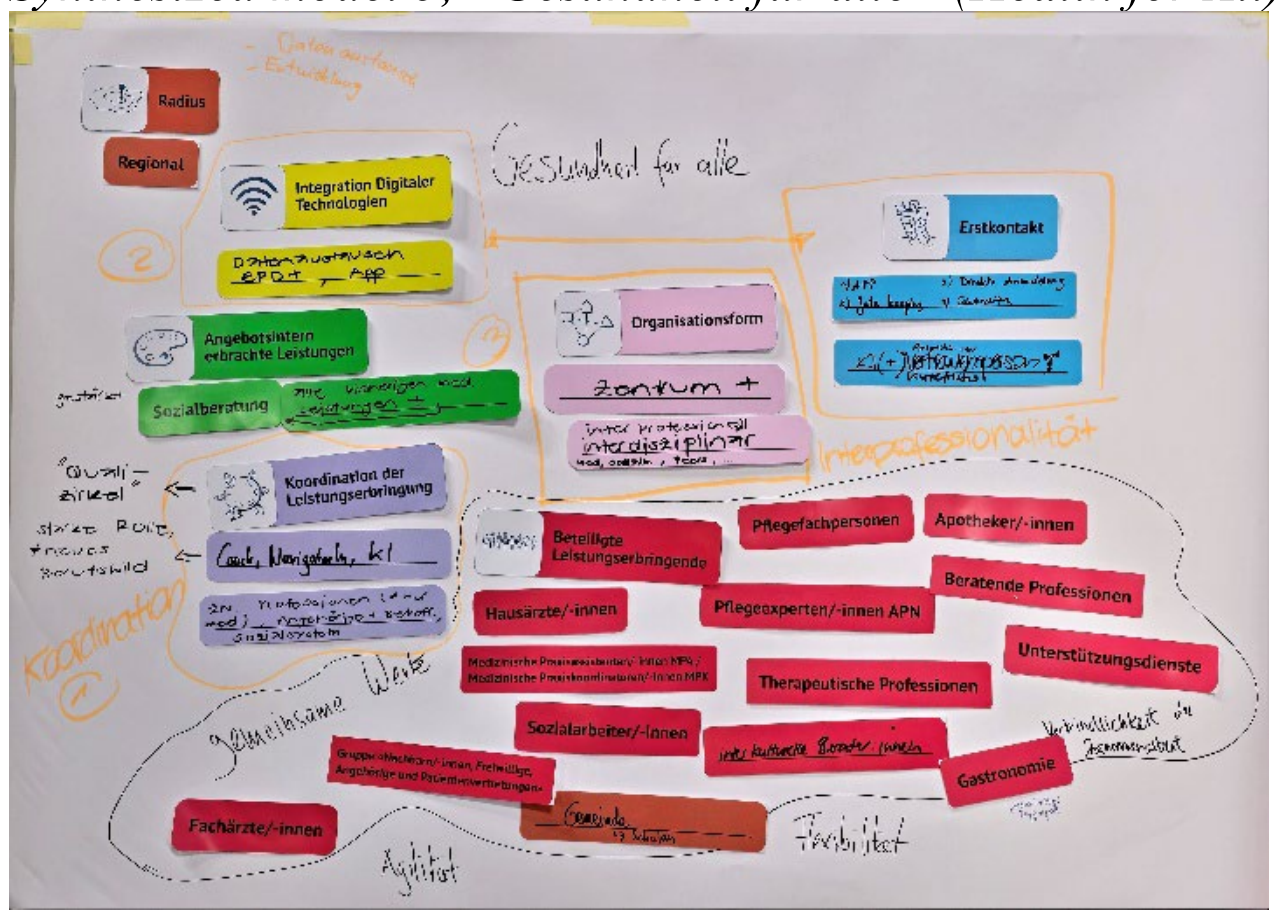
</td>
<td>Regional interprofessional health center integrating health, social, community, and civil-society actors; multiple low-threshold access routes, including direct registration, app, telemedicine, and potentially AI-supported triage; a strong coordinator/navigator role for complex and longer-term care; comprehensive medical and social services, with strengthened social counselling and community involvement; flexible participation of providers depending on context; shared values, commitment, and agility in collaboration; and digital technologies supporting data exchange and coordination.

Proposed Action Areas: coordination of care across health, social, patient/family, and community actors; further development of shared digital data exchange (incl. An app and electronic patient records); and stronger interdisciplinary and interprofessional collaboration.</td>
</tr>
</table>

**Workshop II: X-Curve exercise**	*Group 1 X-Curve poster for the Action Area "Local Support."*<br>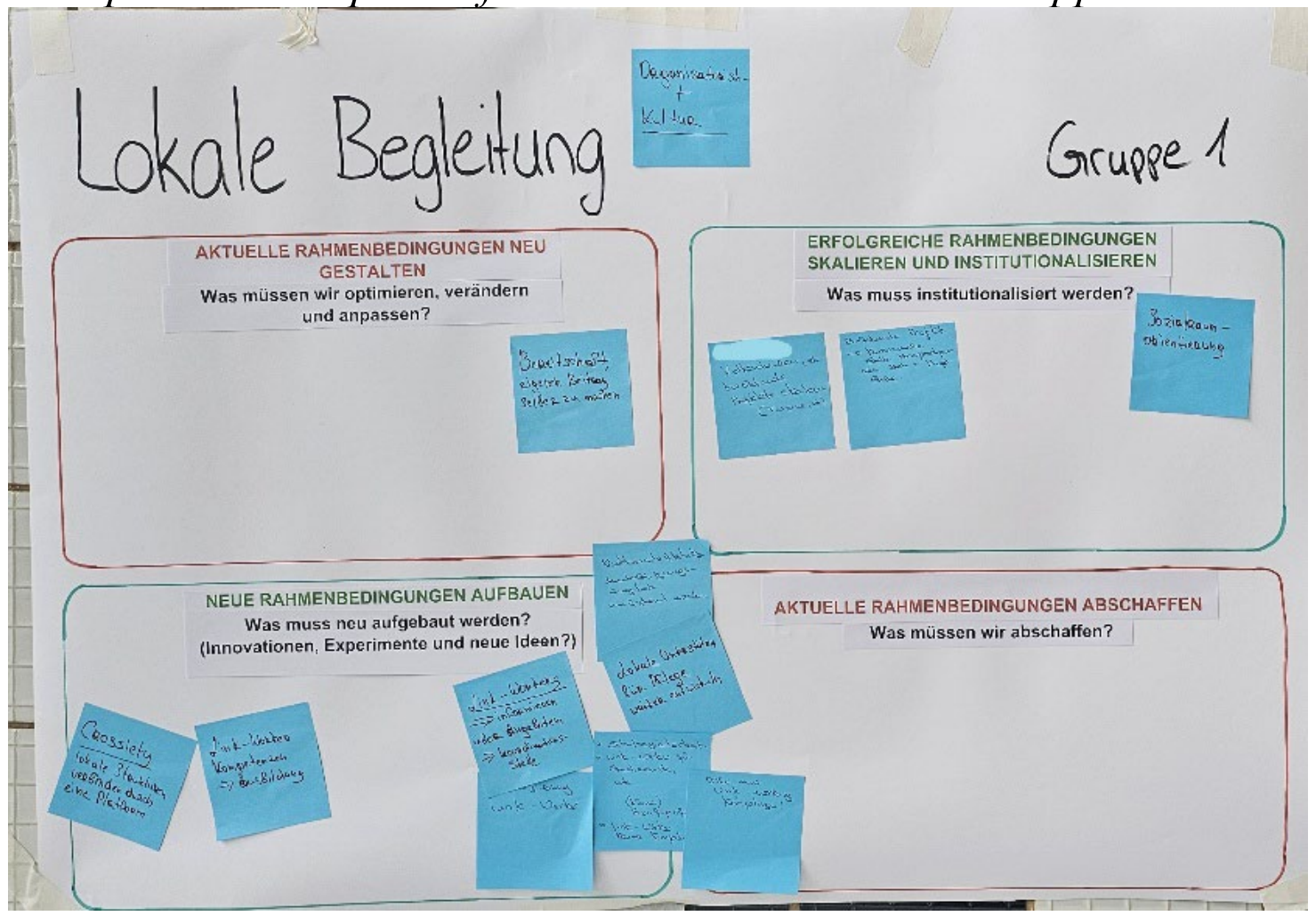<br><br>*Organization names have been blurred to preserve anonymity.*	Improve: willingness to make one's own contribution.<br>Abolish: no specific elements identified.<br>Introduce: connect local structures through a platform; develop link-worker competencies through training; link workers to provide information about available services and act as a coordinating point; further develop local support for care.<br>Institutionalize: social-space orientation; strengthen existing projects.	Identified framework conditions and activities required for implementation
**Workshop II: profession-specific action planning**	*Action planning by the "Coordination and Administration" group.*<br>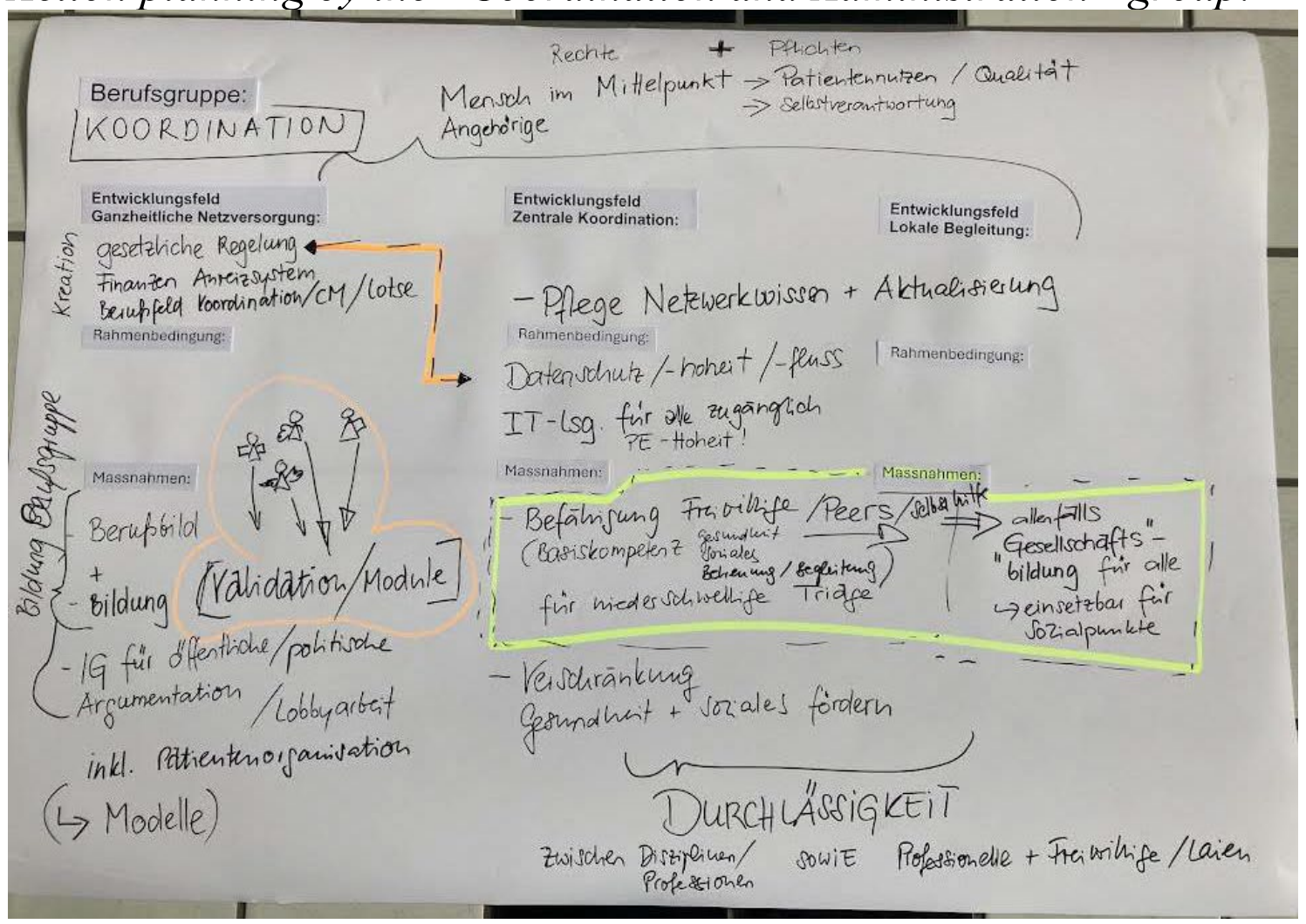 	Action Areas 1–2 – Networked Care / Central Coordination<br>Framework condition: development of a central coordinator professional role<br>Activities proposed by the group: establish an interest group; conduct advocacy/lobbying; develop the professional profile; and develop modular additional training leading to a qualification in coordination for trained professionals in nursing, social work, and medical practice assistance.<br><br>Action Area 3 – Local Support<br>Framework condition: standardized preparation of local peers, volunteers, and self-help actors for a "companion" role.<br>Activities proposed by the group: provide low-threshold basic training in health, social issues, and care/support; consider a community/social-service model involving "social points."	Activities were consolidated, prioritized, and incorporated into the draft Action Plan
