# Supplementary material for: Co‐Creation for Health System Innovation: Developing a Model and an Action Plan for Future Primary Care in Switzerland
Source: Health Expect. 2026 Oct 22;29(5):e70927. doi: 10.1111/hex.70927 (PMC13688086; doi:10.1111/hex.70927)
Supplement: Supplementary file 3 [file HEX-29-e70927-s003.docx]

*Co-creation for health system innovation: Developing a model and an action plan for future primary care in Switzerland*

**Supplementary File 1. Workshop Agendas**

**Workshop I Agenda**

| ***Time*** | ***Session*** | ***Process details*** |
| --- | --- | --- |
| 8:45 | **Plenary I** | Plenary session with slides and interactive warm-up and icebreaker activities (*Tell your Story by Means of an Object*). |
| 9:30 (incl. individual break) | **Group work I:** Development of patient-specific future care models | Small-group discussions. Each group worked with one of five Patient Profiles. Session opened with patient representatives sharing experiences. Using Building Blocks, groups co-created future care models. Outputs were visualized on A0 posters and flipcharts and later presented in plenary. |
| 11:30 | Presentation of models | Each group presented its co-created care model using their posters. Presentations focused on key design considerations and were followed by short rounds of questions and feedback from other participants. |
| 12:00 | *Lunch* | |
| 13:00 | **Plenary II** | Plenary session with activation exercise and introduction to Group Work II, including regrouping of participants. |
| 13:15 | **Group work II:** Synthesis of care models | Mixed-group discussions to synthesize patient-specific models into a shared vision with core elements, patient-specific branches, and three action areas. |
| 15:00 | *Coffee break* | |
| 15:15 | **Plenary III** | Group pitches of synthesized models followed by plenary reflection on outcomes, key action areas, and outlook toward Workshop II. |
| 16:00 | *Next steps and closing* | |

**Workshop II Agenda**

| ***Time*** | ***Session*** | ***Process details*** |
| --- | --- | --- |
| 8:45 | **Plenary I** | Overview of Workshop II goals and program, warm-up activity, and recap of Workshop I. Presentation of the synthesized model and action areas with video inputs. |
| 9:45 (incl. individual break) | **Group work I:** Framework conditions | Small-group discussions using the fishbowl method and X-Curve canvases to identify framework conditions needed for implementing the co-created model. |
| 11:40 | **Plenary II** | Individual reflections on framework conditions; graphic recording summary. |
| 12:00 | *Lunch* | |
| 13:00 | **Plenary III** | Introduction to group work II |
| 13:15 | **Group work II:** Activities to improve framework conditions | Small-group discussions in seven professional groups to defined concrete activities measures their profession could initiate to strengthen framework conditions. Gallery walk: groups visited each other’s posters, asked questions, and gave input. Finalization of measures and preparation of short presentations. |
| 15:00 | *Coffee break* | |
| 15:25 | **Plenary IV:** Presentation of measures, next steps, and outlook | Groups presented three key profession-specific activities. All participants then discussed the proposals and rated them via Mentimeter to indicate whether they could fully support, partially support, or not support the activities. Graphic recording summary. |
| 16:20 | *Next Steps and Closing* | |
